# Circadian gene Rev-erbα influenced by sleep conduces to pregnancy by promoting endometrial decidualization via IL-6-PR-C/EBPβ axis

**DOI:** 10.1186/s12929-022-00884-1

**Published:** 2022-11-24

**Authors:** Liyuan Cui, Feng Xu, Chunfang Xu, Yan Ding, Songcun Wang, Meirong Du

**Affiliations:** 1 NHC Key Lab of Reproduction Regulation (Shanghai Institute of Planned Parenthood Research), Hospital of Obstetrics and Gynecology, Fudan University Shanghai Medical College, Shanghai, 200090 China; 2 Shanghai Key Laboratory of Female Reproductive Endocrine Related Diseases, Shanghai, 200090 China; 3 Laboratory for Reproductive Immunology, Hospital of Obstetrics and Gynecology, Fudan University Shanghai Medical College, ZhaoZhou Road 413, Shanghai, 200011 China; 4 State Key Laboratory of Quality Research in Chinese Medicine and School of Pharmacy, Macau University of Science and Technology, Macau, SAR China; 5 Department of Obstetrics and Gynecology, Guangzhou First People’s Hospital, School of Medicine, South China University of Technology, Guangzhou, 510180 China

**Keywords:** Sleep disturbance, Rev-erbα, Decidualization, Pregnancy

## Abstract

**Background:**

Sleep disturbance can cause adverse pregnancy outcomes by changing circadian gene expression. The potential mechanisms remain unclear. Decidualization is critical for the establishment and maintenance of normal pregnancy, which can be regulated by circadian genes. Whether Rev-erbα, a critical circadian gene, affects early pregnancy outcome by regulating decidualization needs to be explored.

**Methods:**

QPCR, western blot and artificial decidualization mouse model were used to confirm the effect of sleep disturbance on Rev-erbα expression and decidualization. The regulatory mechanism of Rev-erbα on decidualization was assessed using QPCR, western blot, RNA-Seq, and Chip-PCR. Finally, sleep disturbance mouse model was used to investigate the effect of therapeutic methods targeting Rev-erbα and interleukin 6 (IL-6) on improving adverse pregnancy outcomes induced by sleep disturbance.

**Results:**

Dysregulation of circadian rhythm due to sleep disturbance displayed abnormal expression profile of circadian genes in uterine including decreased level of Rev-erbα, accompanied by defective decidualization. Rev-erbα could regulate decidualization by directly repressing IL-6, which reduced the expression of CCAAT/enhancer-binding protein β (C/EBPβ) and its target insulin-like growth factor binding protein 1 (IGFBP1), the marker of decidualization, by inhibiting progesterone receptors (PR) expression. Moreover, deficient decidualization, higher abortion rate and lower implantation number were exhibited in the mouse models with sleep disturbance compared with those in normal mouse. Pharmacological activation of Rev-erbα or neutralization of IL-6 alleviated the adverse effect of sleep disturbance on pregnancy outcomes.

**Conclusions:**

Taken together, Rev-erbα regulated decidualization via IL-6-PR-C/EBPβ axis and might be a connector between sleep and pregnancy outcome. Therapies targeting Rev-erbα and IL-6 might help improving adverse pregnancy outcomes induced by sleep disturbance.

**Supplementary Information:**

The online version contains supplementary material available at 10.1186/s12929-022-00884-1.

## Background

Circadian rhythm regulates multiple behaviors and physiological activities. The suprachiasmatic nucleus (SCN), as the central clock of circadian rhythm, coordinates behavioral and physiological rhythms to the environmental light/dark cycle and synchronizes peripheral clocks through neural and hormonal signals [1]. The basic molecular clockworks generating circadian rhythms are the transcriptional-translational loop consisted of circadian genes. Brain and muscle ARNT-like protein (Bmal1) and circadian locomotor output cycles kaput (Clock) are two main clock genes in this loop, and they drive the transcription of other genes such as period genes (Pers), cryptochrome genes (Crys) and Rev-erbs [2]. Sleep disturbance is a major inductor of circadian rhythm disruption. It has been proposed that sleep disturbance can disrupt external physiological activities such as optical perception time, diet time and sleep activity. However, the circadian rhythm of the central system failed to make accordant adjustment immediately. The disordered circadian rhythm also occurs in the peripheral tissue, and leads to abnormal clock genes expression and hormone secretion [3, 4]. Increasing epidemiological evidences indicate that sleep disturbance is associated with adverse reproductive outcomes such as miscarriage, intrauterine fetal growth restriction and premature birth [3, 5, 6]. Previous studies have suggested that Bmal1, Clock and Per1 knockout mice displayed reproductive abnormality such as irregular estrous cycles, infertility, implantation failure and abortion [7, 8]. Thus, circadian rhythm also plays critical roles in reproduction.

Rev-erbα and Rev-erbβ (also known as nuclear receptor subfamily 1 group D member 1 (NR1D1) and NR1D2) are members of nuclear receptor subfamily 1 group D and play important roles in negative transcriptional-translational loop as transcriptional repressors. The porphyrin heme, as a ligand for Rev-erbs, activates Rev-erbs to repress the transcription of its target genes depending on recruiting nuclear receptor co-repressor—histone deacetylase 3 corepressor complexes [9]. Although 96% of the DNA binding domain of Rev-erbβ is the same as Rev-erbα, their functions are somewhat different [10]. Rev-erbα knockout mice displayed early wakefulness, while Rev-erbβ knockout mice exhibited decreased wakefulness [11, 12], suggesting that Rev-erbα and Rev-erbβ might play complementary roles in regulating sleep–wake cycle. Recent researches indicated that Rev-erbα took participation in the regulation of circadian rhythm, social behavior, lipid metabolism, and cell differentiation [13–16]. Rev-erbα knockout mice displayed pro-inflammatory stimuli and alterations in their circadian locomotor behavior [11, 17]. Sleep disruption decreased Rev-erbα expression in brain and liver [18]. Whether Rev-erbα is a connector between sleep and pregnancy outcomes remains largely unclear.

Decidualization is essential for the establishment and maintenance of pregnancy, characterized with a dramatic morphological and functional differentiation of human endometrial stromal cells (hESCs). It is induced by the increased estradiol and progesterone after ovulation. Progesterone plays critical role during this process by activating the progesterone receptor (PR) [19]. The PR has two major isoforms, PR-A and PR-B, which are encoded by *PGR* gene. The *PGR* knockout mice failed to respond to the artificial decidualization stimulus [20]. The expression of insulin-like growth factor binding protein 1 (IGFBP1) is regarded as a marker of decidualization, and expression of which significantly increased during decidualization [21]. Transcription factors CCAAT/enhancer-binding protein β (C/EBPβ) and forkhead box O 1 (FOXO1) upregulate IGFBP1 expression by binding to its enhancer [22, 23]. Previous researches have demonstrated that PR regulated C/EBPβ expression during decidualization [24]. In addition, the regulation of decidualization is also affected by circadian rhythm genes [25, 26]. Being not only an important circadian clock gene, Rev-erbα is also a transcription factor, while its role in decidualization and establishment and maintenance of pregnancy remain unelusive.

In this study, we first determined if Rev-erbα might be a potential link between sleep disturbance and adverse pregnancy outcomes, and then revealed that Rev-erbα could regulate decidualization. Further, we clarified the potential mechanism of Rev-erbα on decidualization. Finally, the functional regulation of Rev-erbα on adverse pregnancy outcomes in mice with sleep disturbance was investigated.

## Methods

### Mice

All C57 BL/6 mice (6–8 weeks) were purchased from Shanghai SLAC Laboratory Animal Co., Ltd. Mice were bred in a room of 22–25 °C, 40–60% relative humidity, 12 h light-12 h dark cycles with the same time of light-on every day and fed with food and water ad libitum. The mouse vagina was rinsed with physiological saline to detect estrus cycle at nine o'clock every day. The mice with normal estrus cycle were used in the following experiments. For sleep disturbance model, the mice were raised in room of 12 h light-12 h dark cycles with different time of light-on. The time of light-on (referred to ZT0) was advanced 6 h every four days for 3 months. For rhythmic oscillation test, uterus was collected from mice at diestrous phase and frozen on dry ice immediately. For in vivo decidualization, the female mice and vasectomized male mice were caged together at 19:00, and the vaginal plugs were detected at next 7:00, which referred to pseudopregnancy 0.5 days (PE0.5). Unilateral uterine horn was injected with 25 μL sesame oil at PE3.5, and the decidual level was analyzed at PE7.5. For pregnancy outcomes assay, the female mice and male mice were caged together at 19:00, and the vaginal plugs were detected at next 7:00, which referred to embryonic 0.5 days (E0.5). The mice with normal sleep were injected with physiological saline. Some mice with sleep disturbance were injected with 50 mg/kg SR9009 (HY-16989, MedChemExpress) once daily or 10 mg/kg anti-IL6 (504513, Biolegend) every three days. All mice were sacrificed at E13.5 to observe the pregnancy outcomes. All experimental procedures of mice were approved by the Institutional Animal Care and Use Committee at Fudan University.

### Quantitative real-time PCR (QPCR)

Total RNA was extracted from cells or homogenized tissues using TRIzol reagent (T9108, Takara) according to the manufacturer’s instructions. Complementary DNA (cDNA) was synthesized using PrimeScript™ RT Master Mix (RR036, Takara) and then amplified using SYRB Green PCR Master Mix (RR820, Takara) with ABI PRISM 7900 Sequence Detection System (Applied Biosystems, Waltham, MassachusettsMA, USA). *β-Actin* (*Actb*) was used as an internal control to normalize the relative changes in gene expression using the 2^−△△Ct^ method. Human primer sequences for QPCR: *Rev-erbα*, forward 5′-TGGACTCCAACAACAACACAG -3′ and reverse 5′-GATGGTGGGAAGTAGGTGGG-3′; *Rev-erbβ*, forward 5′- TCATGCTTGCGAAGGCTGTAA-3′ and reverse 5′-CGCTTAGGAATACGACCAAACC-3′; *Bmal1*, forward 5′-CATTAAGAGGTGCCACCAATCC-3′ and reverse 5′-TCATTCTGGCTGTAGTTGAGGA-3′; *Clock*, forward 5′-TGCGAGGAACAATAGACCCAA-3′ and reverse 5′-ATGGCCTATGTGTGCGTTGTA-3′; *IGFBP1*, forward 5′-CGAAGGCTCTCCATGTCACCA-3′ and reverse 5′-TGTCTCCTGTGCCTTGGCTAAAC-3′; *PGR*, forward 5′-TGTATTTGTGCGTGTGGGTG-3′ and reverse 5′-TACAGCCCATTCCCAGGAAG-3′; *C/EBPβ*, forward 5′-CTTCAGCCCGTACCTGGAG -3′ and reverse 5′-GGAGAGGAAGTCGTGGTGC-3′. Mouse primer sequences for QPCR: *Rev-erbα*, forward 5′-TACATTGGCTCTAGTGGCTCC-3′ and reverse 5′-CAGTAGGTGATGGTGGGAAGTA-3′; *Rev-erbβ*: forward 5′- TGAACGCAGGAGGTGTGATTG-3′ and reverse 5′-GAGGACTGGAAGCTATTCTCAG-3′; *Bmal1*: forward 5′-GGCGTCGGGACAAAATGAAC-3′ and reverse 5′-TCTTCCCTCGGTCACATCCT-3′; *Dtprp*: forward 5′-AAGAATGCCCTTCAGCGAGC-3′ and reverse 5′-AGCTGGTGGGTTTGTGACAT-3′; *Wnt4*: forward 5′-AGACGTGCGAGAAACTCAAAG-3′ and reverse 5′-GGAACTGGTATTGGCACTCCT-3′; *Bmp2*: forward 5′-GGGACCCGCTGTCTTCTAGT-3′ and reverse 5′-TCAACTCAAATTCGCTGAGGAC-3′, *IL-6*, forward 5′- ATCCAGTTGCCTTCTTGGGACTGA-3′ and reverse 5′-TAAGCCTCCGACTTGTGAAGTGGT-3′; *PGR*, forward 5′-CTCCGGGACCGAACAGAGT-3′ and reverse 5′-ACAACAACCCTTTGGTAGCAG-3′.

### Human samples

Human endometrial tissues during secretory phase were collected from women with regular menstrual cycles who did not have underlying endometrial abnormalities and did not receive exogenous steroidal hormones therapy for three months preceding biopsy collection. Human decidual tissues (gestational age: 6–12 weeks) were obtained from healthy pregnancies who were aged between 22 and 40 and artificially terminated for non-medical reasons or miscarriages who were diagnosed as unexplained abortion excluding chromosomal defects, genetic abnormalities, infection, endocrine and other factors. All participants were required to complete the questionnaire of patients pittsburgh sleep quality index (PSQI). Participants with PSQI ≤ 5 were considered to have normal sleep, Participants with PSQI > 5 were considered to have sleep disturbance. Written informed consent was obtained from all participants. All performances were approved by Human Research Ethics Committee of the Obstetrics and Gynecology Hospital of Fudan University.

### Cell culture and treatment

Human endometrial tissues were digested with 1.0 mg/mL collagenase IV (C5138, Sigma-Aldrich) to obtain hESCs and they were cultured in complete medium (Dulbecco’s modified Eagle’s medium/F-12 (DMEM/F12 supplemented with 10% fetal bovine serum, 100 U/mL penicillin and 100 μg/mL streptomycin) as described previously [27]. Human decidual stromal cells (hDSCs) were separated from decidual tissues after digestion with 1.0 mg/mL collagenase IV (C5138, Sigma-Aldrich) and 150 U/mL DNase I in DMEM/F12 and density gradient centrifugation with percoll, as described previously [28].

Mouse endometrial stromal cells (mESCs) were isolated from mouse uteruses during diestrous phase followed by prior studies [29, 30]. Briefly, mouse uteruses were cut into 2–3 mm pieces and digested with 6 mg/ml dispase II (17105041, Gibco) and 25 mg/ml trypsin (T8150, Solarbio) for 1 h at 4 °C on a shaker, 1 h at room temperature without shaking, and 30 min at 37 °C without shaking, after which tissues were washed twice with hank's balanced salt solution. The remaining tissues were digested with 0.5 mg/ml collagenase at 37 °C for 30 min, and then filtered through 70 μm filter to obtain stromal cells. The stromal cells were cultured in complete medium for 1 h, and then the mixed complete medium was replaced with fresh complete medium.

For si-RNA transfection, h/mESCs were dealt with *Rev-erbα*/*PGR*/*C/EBPβ*-specific siRNA (Si-RNA for hESCs: si-*Rev-erbα*: CATGTCCTATGAACATGTA; si-PGR: GCACCTGATCTAATACTAA; si–*C/EBPβ*: CCATGGAAGTGGCCAACTT. Si-RNA for mESCs: si-*Rev-erbα*: GTACAAACGGTGTCTGAAA; si-*PGR*: CCATGTAAAGAGCACCATA; si–*C/EBPβ*: GAGCGACGAGTACAAGATG) for 20 h using transfection reagent (L3000015, Invitrogen) according to the manufacturer’s instructions. For in vitro decidualization, hESCs were treated with 1 mM MPA and 0.2 mg/mL cAMP (T1418, Topscience, Shanghai, China) in complete medium for 48 h; mESCs were treated with 10 nM estradiol (E2) (T1048, Topscience, Shanghai, China) and 1 μM progesterone (P4) (T0478, Topscience, Shanghai, China) in complete medium for 72 h. For IL-6 treatment, h/mESCs were dealt with IL-6 (200-06-5, PeproTech; 216–16, PeproTech) with indicated concentrations for 4 h before in vitro decidual treatment. For antibody neutralizing or inhibitor tests, h/mESCs were treated with 2.5 μg/mL anti-IL-6 (501125, biolegend; 504513, Biolegend) or 100 mg /mL Tocilizumab (IL-6R inhibitor) (HY-P9917, MedChemExpress) for 4 h before si-RNA transfection.

### Western blot

Western blot was performed as described previously [28]. The primary antibodies were as follows: anti-IGFBP1 (ab180948, Abcam), anti-Rev-erbα (sc-393215, Santa Cruze), anti-β-Actin (ab179467, Abcam), anti-β-Tubulin (ab179513, Abcam), anti-PR (human, 8757, Cell Signaling Technology), anti-C/EBPβ (ab32358, Abcam); anti-IL-6 (human, ab233706, Abcam), anti-IL-6R (human, ab222101, Abcam), anti-PR (mouse, ab133526, Abcam), anti-IL-6 (mouse, ab229381, Abcam), anti-IL-6R (mouse, ab300581, Abcam), anti-Wnt4 (sc-376279, Santa Cruze). β-Tubulin and β-Actin were used as internal standards.

### RNA-Seq

Total RNA was extracted from hESCs treated with si-RNA transfection and in vitro decidualization using TRIzol reagent according to the manufacturer’s instructions. mRNA was enriched from total RNA and then constructed a cDNA library, which was sequenced on the BGISEQ-500 sequencing platform (BGI-shenzhen Technology Co., Ltd).

### Chromatin immunoprecipitation-polymerase chain reaction (ChIP-PCR)

HESCs were fixed and cross-linked with 1% formaldehyde for 10 min at room temperature. And then they were sonicated into fragments of 200–700 bp after terminated cross-linking with 125 mM glycine. Sonicated products were divided into two groups, one group was used as the input control. Another group was incubated with antibodies (anti-Rev-erbα, 13418, Cell Signaling Technology; anti-IgG, ab172730, Abcam) overnight at 4 ℃, and then incubated with protein A/G immunomagnetic beads to obtain protein-DNA complex. After DNA was purified, qPCR was used to identify the enriched genes. Primers were as follows: *IL-6*, forward 5′-TGCACTTTTCCCCCTAGTTG-3′ and reverse 5′-TCATGGGAAAATCCCACATT -3′; *IL-6R*, forward 5′-GAGGGCAGAGGCACTTACTG-3′ and reverse 5′-AGTTGCCCAACTCTTCCAGA-3′; Negative, forward 5′-TGTGTGGAGCCAACAGTCTC-3′ and reverse 5′-CAGAAAAGCCCAGATGGAAA-3′.

### Immunofluorescence and hematoxylin–eosin (HE) staining

Paraffin-embedded section of decidual tissues were dewaxed using dimethylbenzene and rehydrated in ethanol at different concentrations (100%, 95%, 90%, 80%, 70% and 50%). For immunofluorescence, the sections were blocked with 10% donkey serum after antigen retrieval using citrate sodium solution, and then they were incubated with primary antibodies (anti- Rev-erbα (sc-393215, Santa Cruze); anti-Vimentin (ab92547, Abcam), anti-Wnt4 (sc-376279, Santa Cruze)) overnight at 4 ℃. The sections were incubated with secondary antibodies for 2 h at room temperature after washed three times with tris-buffered saline (TBS) (10 min each), followed by 4′,6-diamidino-2-phenylindole (DAPI) staining. Mean gray value was calculated using ImageJ software. Relative mean gray value = mean gray value of cells /the mean value of mean gray value of cells from human/mouse with normal sleep. For hematoxylin–eosin (HE) staining, the sections were stained with hematoxylin solution for 5 min, and then washed with ultrafiltration water for 5 s. Next, the sections were stained with eosin solution for 3 min and dehydrated in ethanol at different concentrations (50%, 70%, 80%, 90%, 95% and 100%) and dimethylbenzene in turn. The slides were sealed with mounting medium and taken pictures using a fluorescence microscope.

### Statistical analysis

GraphPad Prism version 7 was used to analyze the statistical difference. A Student’s tail *t*-test was performed to determine the statistical significance of differences between two groups. P < 0.05 was considered as statistically significant difference. Data were showed as mean ± standard error of the mean (SEM).

## Results

### Dysregulated circadian gene profile was observed in mice and human with sleep disturbance

**Fig. 1:**
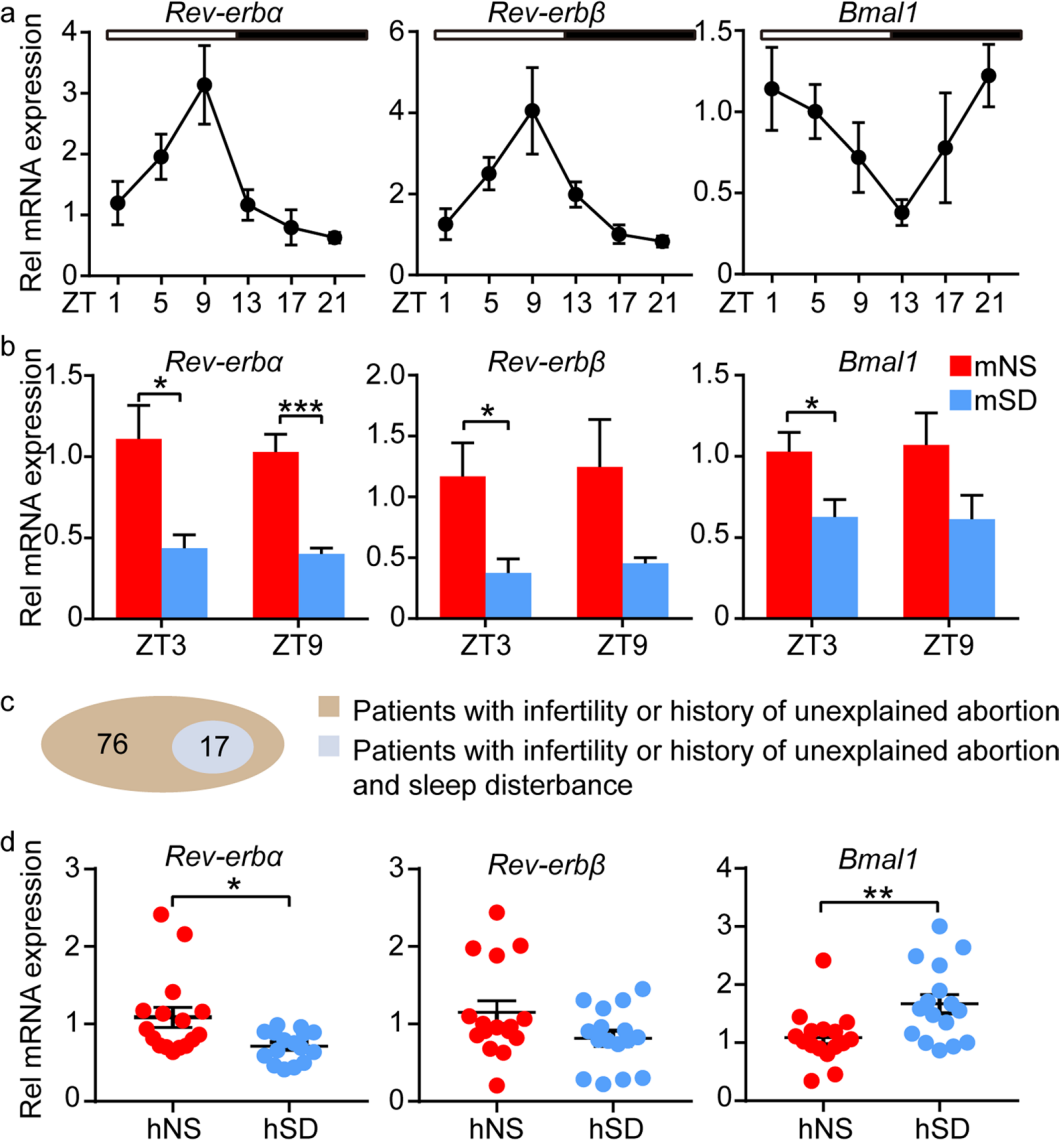
Dysregulated circadian genes in mice and human with circadian rhythm disruption. **a** The relative mRNA level of clock genes (*Rev-erbα*, *Rev-erbβ*, *Bmal1*) in uterus of mice with normal sleep in 24 h. White box represented light-on time. Black box represented light-off time. **b** The relative mRNA level of clock genes in uterus of mice with normal or sleep disturbance at ZT3 (three hours after light-on) and ZT9. **c** the number of patients with infertility or history of unexplained abortion and those with sleep disturbance. **d** The relative mRNA level of clock genes in ESCs of human with normal or sleep disturbance. mNS represented mouse with normal sleep. mSD represented mouse with sleep disturbance. hNS represented human with normal sleep. hSD represented human with sleep disturbance. The time of light on referred to ZT0. Data represented Mean ± SEM. Statistical analysis was performed using Student’s *t*‐test. *P < 0.05, **P < 0.01, ***P < 0.001

Rev-erbs and Bmal1 are main circadian genes, whose expression in uterus of mice with normal sleep displayed rhythmic oscillation (Fig. 1a). Rev-erbs could directly inhibit the expression of Bmal1, so the rhythmic oscillation of *Rev-erbs* in uterus was in antiphase to that of *Bmal1*, which are similar to that in liver (Fig. 1a, Additional file 1: Fig. S1). The expression of Rev-erbs and Bmal1, especially Rev-erbα, were significantly decreased in uterine tissues and ESCs of mice with sleep disturbance compared to those with normal sleep (Fig. 1b, Additional file 2: Fig. S2a, b). We also found that 22.37% (17/76 patients) patients with infertility or miscarriage appear symptoms of sleep disturbance (Fig. 1c, Table1). And the expression of Rev-erbα in ESCs of women with sleep disturbance was decreased compared to that with normal sleep (Fig. 1d, Additional file 2: Fig. S2c). Therefore, sleep disturbance could alter the expression of Rev-erbα in ESCs.

**Table 1:** Characteristics of recruited participants

Subjects	Normal sleep	Sleep disruption	*P* value
Number	59	17	–
Age range (years)	25–41	23–39	–
Age mean^a^	32.85 ± 0.57	30.59 ± 0.75	ns
Childbearing history (n(%))	13 (22.03%)	5 (29.41%)	–
Infertility (n(%))	6 (10.17%)	2 (11.76%)	–
Abortion (n(%))	53 (89.83%)	15 (88.24%)	–
Number of abortion^b^	2.21 ± 1.29	2.33 ± 0.94	ns
Treatment history	–	–	–

^a^Mean ± SEM; ^b^Mean ± standard deviation (SD)

### Rev-erbα regulates endometrial decidualization

Endometrial decidualization is essential for successful pregnancy. To clarify the correlation between Rev-erbα and decidualization, we first compared the spatiotemporal expression of Rev-erbα in murine uterine tissue at different gestation period (from E0.5 to E7.5). Robust Rev-erbα expression was detected in luminal and glandular epithelial cells on E0.5 and E3.5, with weaker signal in stromal cells. In rodents, embryo implantation occurs at midnight of E3.5, after which the stromal cells initiated the decidualization. Previous study proved the expression of Wnt4, a decidual marker in uterus of mouse, was localized to the sub-luminal stromal cells immediately surrounding the implanting blastocyst on E4.5 [31]. We found that Rev-erbα expression was significantly increased in sub-luminal stromal cells following implantation on E4.5, and evident signals were also detected in luminal and glandular epithelial cells. The stromal cells differentiated to form an avascular primary decidual zone on the afternoon of E4.5. So E5.5 is a key time point in the decidualization process. Rev-erbα expression was detected throughout the stromal bed on E5.5, and it was mainly observed in the mesometrial decidual beds on E6.5 and E7.5 (Additional file 3: Fig. S3a). The protein level of Rev-errbα in murine uterine tissues was significantly increased from E4.5 to E7.5 compared to that on E0.5 and E3.5 (Additional file 3: Fig. S3b). Moreover, Rev-erbα expression in hDSCs was higher than that in hESCs (Additional file 4: Fig. S4a). Therefore, Rev-erbα might be involved in decidualization.

**Fig. 2:**
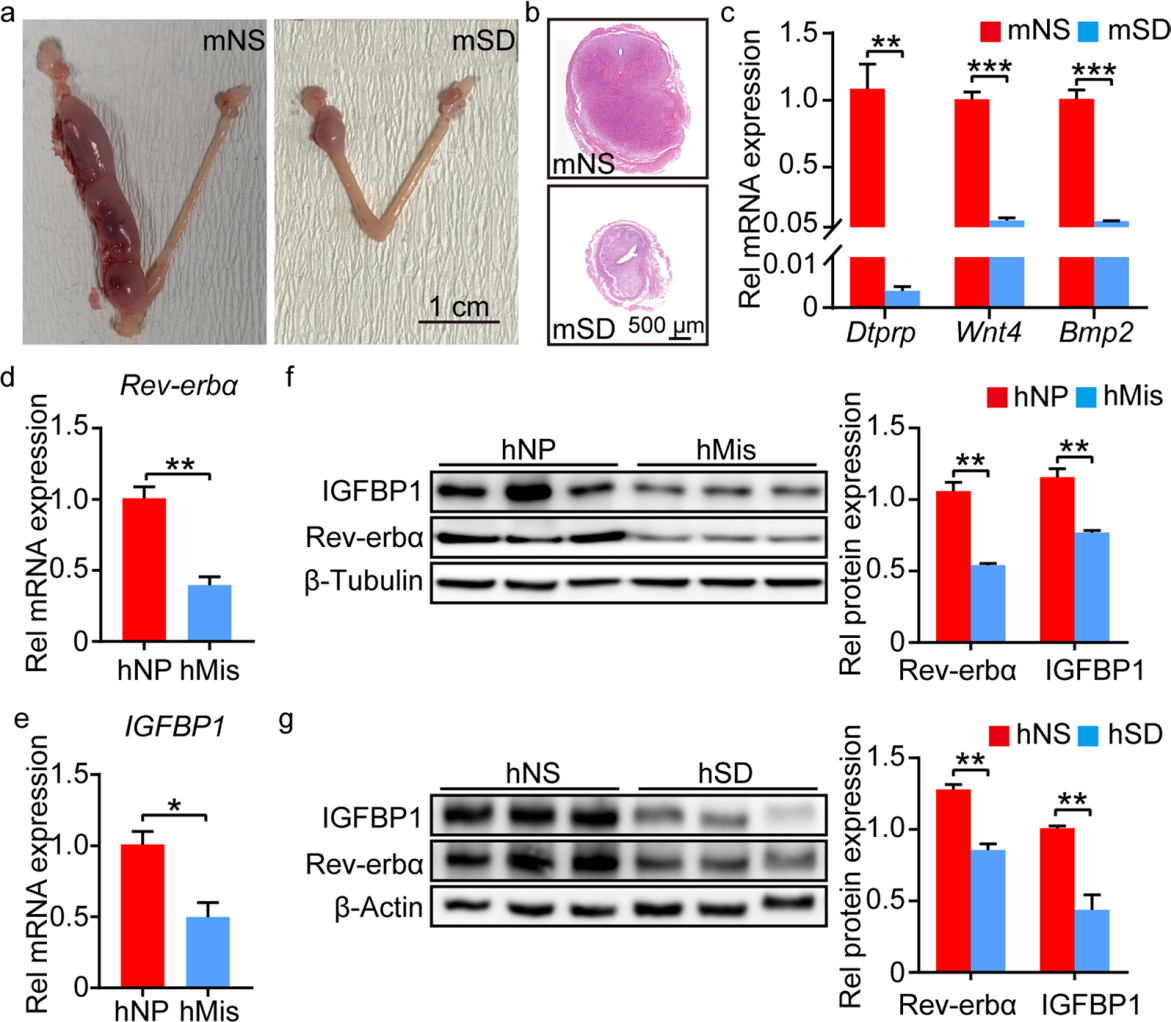
Deficient decidualization in mice and human with sleep disturbance.** a** The representative picture of uterus from mice with normal or sleep disturbance after artificial decidualization. **b** HE staining for cross section of uterus from mice with normal or sleep disturbance after artificial decidualization. **c** The relative mRNA level of decidualization markers (*Dtprp*, *Wnt4*, *Bmp2*) in oil-injected lateral uterus of mice with normal or sleep disturbance. **d**, **e** The relative mRNA level of *Rev-erbα* and *IGFBP1* in DSCs of human normal pregnancies with normal sleep and miscarriages with sleep disturbance. **f** The protein level of Rev-erbα and IGFBP1 in DSCs of human normal pregnancies with normal sleep and miscarriages with sleep disturbance. Relative protein levels were normalized to β-Tubulin. **g** The protein level of Rev-erbα and IGFBP1 in hESCs of human with normal or sleep disturbance after in vitro decidualization. Relative protein levels were normalized to β-Actin. mNS represented mouse with normal sleep. mSD represented mouse with sleep disturbance. hNS represented human with normal sleep. hSD represented human with sleep disturbance. hNP represented human with normal pregnancy and normal sleep. hMis represented human with miscarriage and sleep disturbance. Data represented Mean ± SEM. Statistical analysis was performed using Student’s *t*‐test. *P < 0.05, **P < 0.01, ***P < 0.001

Downregulated Rev-erbα expression was observed in ESCs of mice with sleep disturbance (Fig. 1). As we expected, the decidualization in mice with sleep disturbance was defective compared to that with normal sleep, as confirmed by the expression of mouse decidual markers, *Dtprp*, *Wnt4* and *Bmp2* in decidual tissues (Fig. 2a-c). The expression of Rev-erbα and Wnt4 were decreased in Vimentin^+^ DSCs of mice with sleep disturbance compared to those with normal sleep after in vivo decidualization (Additional file 5: Fig. S5). Deficient decidualization could cause adverse pregnancy outcomes such as miscarriage. We observed dysregulated expression profile of clock genes in hDSCs from patients of miscarriage with sleep disturbance, but not in hDSCs from normal pregnancy with normal sleep (Additional file 4: Fig. S4b, c). The decreased expression of Rev-erbα and IGFBP1 were also shown in hDSCs from patients of miscarriage with sleep disturbance compared to those from normal pregnancy with normal sleep (Fig. 2d–f). Moreover, the expression of Rev-erbα and IGFBP1 was also reduced in hESCs from human with sleep disturbance compared to that from normal sleep after in vitro decidualization (Fig. 2g). These results suggested that Rev-erbα might play important role in the regulation of decidualization.

**Fig. 3:**
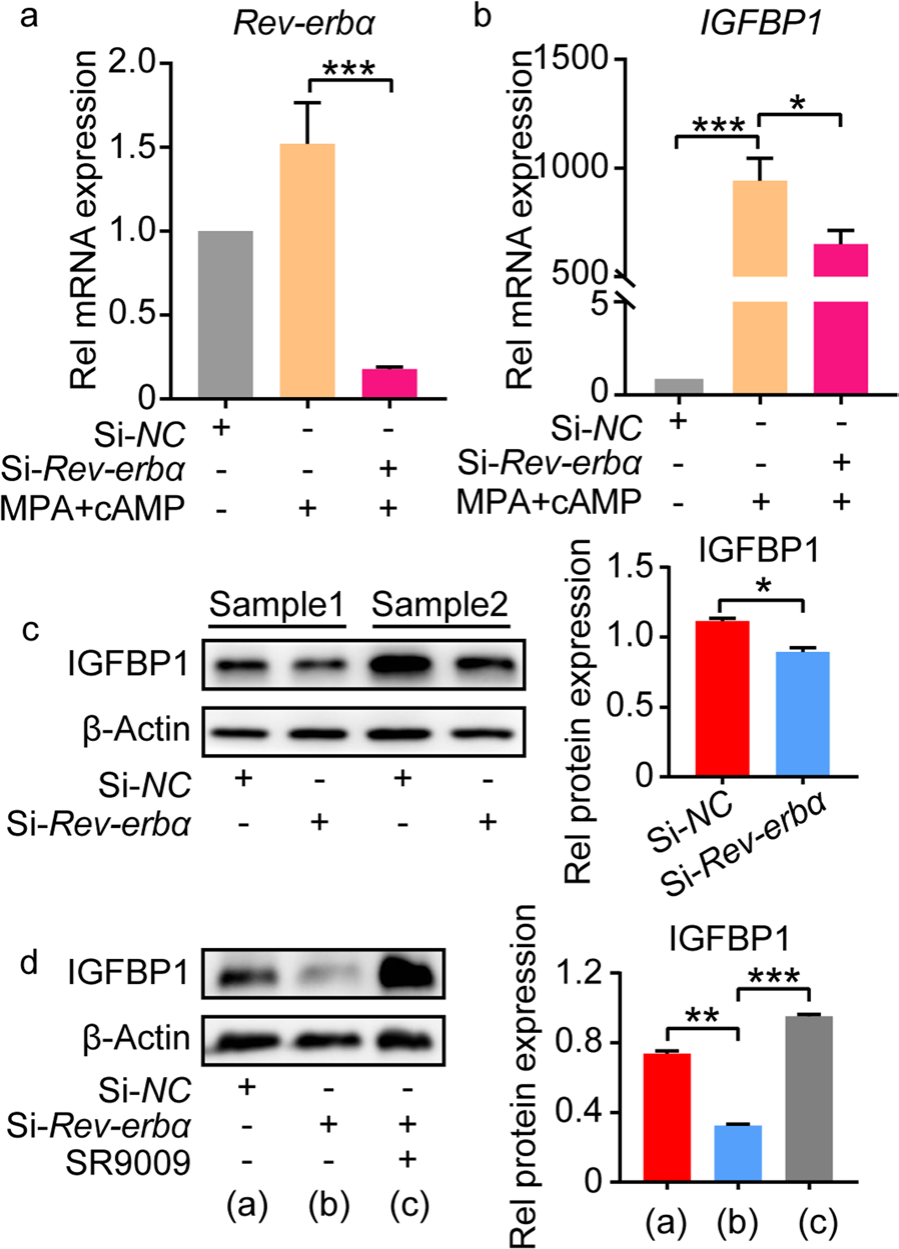
*Rev-erbα* knockdown induced defective decidualization in hESCs. **a**, **b** The relative mRNA level of *Rev-erbα* and *IGFBP1* in hESCs with or without *Rev-erbα* knockdown. **c** The protein level of Rev-erbα and IGFBP1 in hESCs with or without *Rev-erbα* knockdown. Relative protein levels were normalized to β-Actin. **d** SR9009 alleviated the defective decidualization induced by *Rev-erbα* knockdown in hESCs. Relative protein levels were normalized to β-Actin. Data represented Mean ± SEM. Statistical analysis was performed using Student’s *t*‐test. *P < 0.05, **P < 0.01, ***P < 0.001

To further confirm the regulatory role of Rev-erbα in decidualization, we analyzed the decidualization of ESCs with *Rev-erbα* knockdown. The mRNA level of *Rev-erbα* remarkably decreased in hESCs with si-*Rev-erbα* transfection (Fig. 3a). The decreased expression of IGFBP1 and Wnt4 was also observed in hESCs and mESCs with *Rev-erbα* knockdown compared to the control, respectively (Fig. 3b, c, Additional file 6: Fig. S6). SR9009, an agonist of Rev-erbα, reversed the defective decidualization caused by *Rev-erbα* knockdown both in hESCs and mESCs (Fig. 3d, Additional file 6: Fig. S6). These results suggested that Rev-erbα played important roles in decidualization.

### Rev-erbα regulated decidualization via IL-6-PR-C/EBPβ pathway

**Fig. 4:**
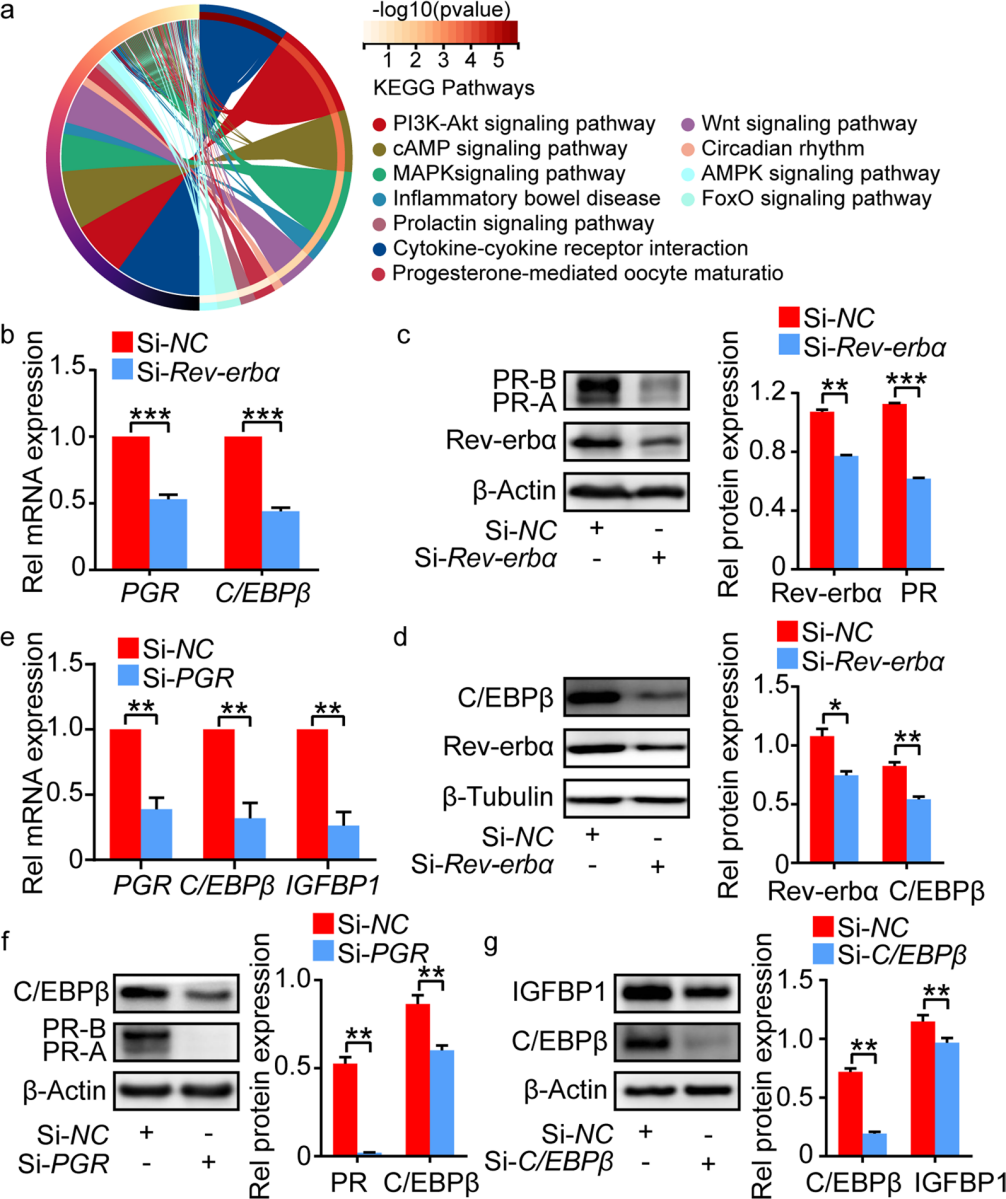
*Rev-erbα* knockdown downregulated PR and C/EBPβ expression in hESCs. **a** KEGG analysis results of differentially expressed genes between hESCs without *Rev-erbα* knockdown and that with *Rev-erbα* knockdown. **b** The relative mRNA level of *PGR* and *C/EBPβ* in hESCs with or without *Rev-erbα* knockdown. **c** and **d** The protein level of PR and C/EBPβ in hESCs with or without *Rev-erbα* knockdown. Relative protein levels were normalized to β-Actin (in **c**) or β-Tubulin (in **d**). **e** The relative mRNA level of *PGR*, *C/EBPβ* and *IGFBP1* in hESCs with or without *PGR* knockdown. **f** The protein level of C/EBPβ in hESCs with or without *PGR* knockdown. Relative protein levels were normalized to β-Actin. **g** The protein level of IGFBP1 in hESCs with or without *C/EBPβ* knockdown. Relative protein levels were normalized to β-Actin. Data represented Mean ± SEM. Statistical analysis was performed using Student’s *t*‐test. *P < 0.05, **P < 0.01, ***P < 0.001

To further investigate the regulatory mechanism of Rev-erbα on decidualization, we screened the differentially expressed genes between hESCs with and without *Rev-erbα* knockdown. The differentially expressed genes enriched in decidualization related and progesterone related signaling pathways by Kyoto Encyclopedia of Genes and Genomes (KEGG) analysis (Fig. 4a). We further confirmed that PR expression was significantly decreased in hESCs and mESCs with *Rev-erbα* knockdown compared to those without *Rev-erbα* knockdown (Fig. 4b, c, Additional file 7: Fig. S7a). C/EBPβ is a critical molecular in decidualization regulated by PR, and IGFBP1 and Wnt4 are two targets of it [23, 24, 32]. Its expression was downregulated in hESCs and mESCs with *Rev-erbα* knockdown or *PGR* knockdown (Fig. 4b and d–f, Additional file 7: Fig. S7a and b). To determine whether PR-C/EBPβ signal participates in the regulation of decidualization, the decidual marker was detected in ESCs with *PGR* or *C/EBPβ* knockdown. As expected, knockdown of *PGR* or *C/EBPβ* could decrease IGFBP1 and Wnt4 expression in hESCs and mESCs during in vitro decidualization, respectively (Fig. 4e and g, Additional file 7: Fig. S7c). These findings suggested that Rev-erbα could regulate decidualization by PR-C/EBPβ signal pathway.

**Fig. 5:**
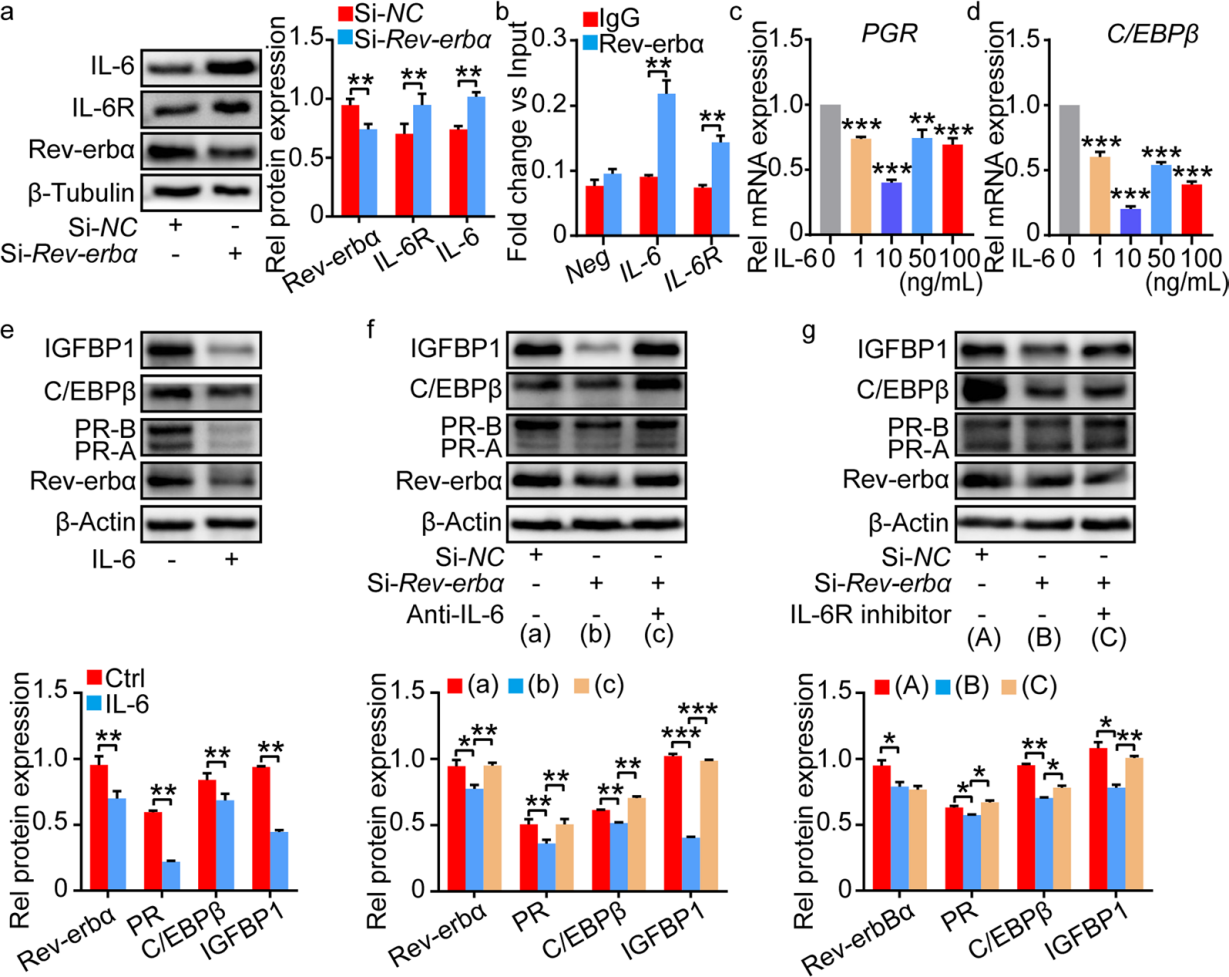
Rev-erbα regulated decidualization via IL-6-PR-C/EBPβ axis in hESCs. **a** The protein level of IL-6 and IL-6R in hESCs with or without *Rev-erbα* knockdown. Relative protein levels were normalized to β-Tubulin. **b** Chip-PCR assay showing recruitment of Rev-erbα to IL-6 and IL-6R promoter in hESCs. **c**, **d** The relative mRNA level of *PGR* and *C/EBPβ* in hESCs stimulated with different concentrations of IL-6. **e** The protein level of PR, C/EBPβ and IGFBP1 in hESCs with IL-6 stimulation. Relative protein levels were normalized to β-Actin. **f** IL-6 neutralized antibody (anti-IL-6) reversed the decreased PR, C/EBPβ and IGFBP1 expression in hESCs with *Rev-erbα* knockdown. Relative protein levels were normalized to β-Actin. **g** IL-6R inhibitor alleviated the decreased PR, C/EBPβ and IGFBP1 expression in hESCs with *Rev-erbα* knockdown. Relative protein levels were normalized to β-Actin. Data represented Mean ± SEM. Statistical analysis was performed using Student’s *t*‐test. *P < 0.05, **P < 0.01, ***P < 0.001

Rev-erbα, a transcription factor, was reported to directly inhibit IL-6 expression in colitis [17]. We also observed increased levels of IL-6 and IL-6 receptor (IL-6R) in hESCs and mESCs after *Rev-erbα* knockdown (Fig. 5a, Additional file 8: Fig. S8a). To determine whether Rev-erbα regulate PR-C/EBPβ signal via suppression of IL-6, we first tested the recruitment of Rev-erbα to *IL-6* and *IL-6R* promoter in hESCs by using Chip-PCR assay. The result in Fig. 5b showed that IL-6 and IL-6R were the direct targets of Rev-erbα. IL-6 also remarkably decreased the expression of PR, C/EBPβ and IGFBP1 or Wnt4 in hESCs or mESCs (Fig. 5c–e, Additional file 8: Fig. S8b). These results suggested that IL-6 could restrain decidualization by controlling the expression of PR and C/EBPβ. In addition, IL-6 neutralized antibody reversed the effect of *Rev-erbα* knockdown on PR, C/EBPβ and IGFBP1 or Wnt4 expression in hESCs or mESCs (Fig. 5f, Additional file 8: Fig. S8c). IL-6R inhibitor also displayed similar beneficial properties against decreased PR, C/EBPβ and IGFBP1 expression in hESCs with *Rev-erbα* knockdown (Fig. 5g). Therefore, we speculated that Rev-erbα could regulate decidualization via IL-6-PR-C/EBPβ axis.

### Activation of Rev-erbα or neutralization of IL-6 alleviated defective decidualization and early pregnancy loss in mice induced by sleep disturbance

**Fig. 6:**
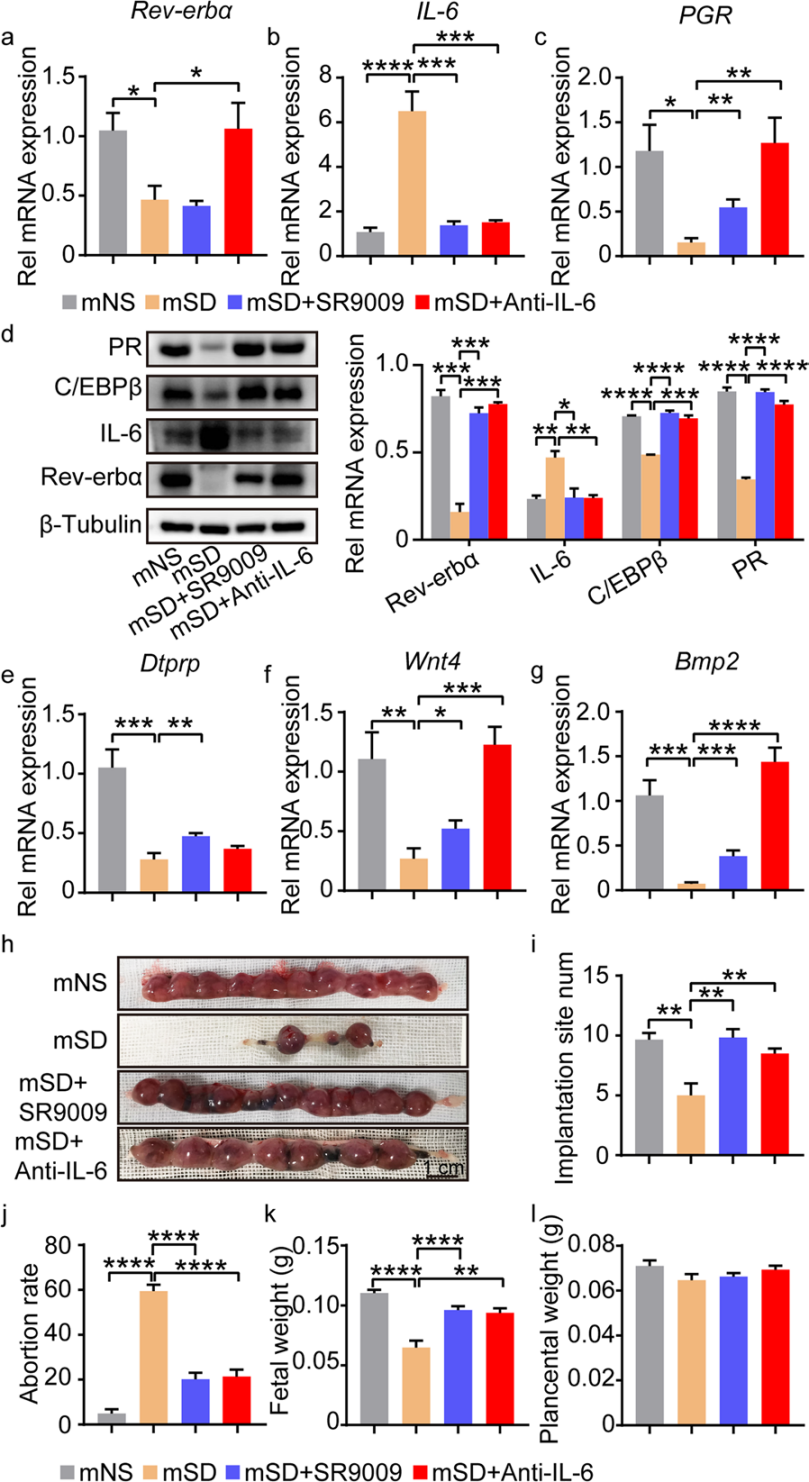
SR9009 and IL-6 neutralized antibody alleviated the effect of circadian rhythm disruption on decidualization and pregnancy outcome in mice. **a–c** Relative mRNA level of *Rev-erbα*, *IL-6* and *PGR* in DSCs from control mice and sleep disturbance mice with SR9009 or IL-6 neutralized antibody treatment. **d** The protein level of PR, C/EBPβ, IL-6 and Rev-erbα in DSCs from control mice and sleep disturbance mice with SR9009 or IL-6 neutralized antibody treatment. Relative protein levels were normalized to β-Tubulin. **e–g** The Relative mRNA level of *Dtprp*, *Wnt4* and *Bmp2* in DSCs from control mice and sleep disturbance mice with SR9009 or IL-6 neutralized antibody treatment. **h** The representative pictures of pregnancy outcomes of control mice and sleep disturbance mice with SR9009 or IL-6 neutralized antibody treatment. **i** The number of implantation site, abortion rate, fetal weight and placental weight of control mice and sleep disturbance mice with SR9009 or IL-6 neutralized antibody treatment. mNS represented mouse with normal sleep. mSD represented mouse with sleep disturbance. Data represented Mean ± SEM. Statistical analysis was performed using Student’s *t*‐test. **P < 0.01, ***P < 0.001

The in vitro experiments suggested that sleep disturbance could inhibit the expression of Rev-erbα, causing deficient decidualization via IL-6/IL-6R-PR-C/EBPβ axis. We then further investigated whether these regulatory relationships also existed in vivo, which might affect pregnancy outcome. As expected, decreased Rev-erbα expression was observed in decidual tissues from mice with sleep disturbance compared with those with normal sleep, accompanied by increased IL-6 and decreased PR and C/EBPβ expression (Fig. 6a–d). Moreover, the decidualization markers were decreased in mice with sleep disturbance compared to those with normal sleep (Fig. 6e–g). Importantly, the implantation number was decreased and abortion rate was increased in mice with sleep disturbance (Fig. 6h–j). The fetal weight was also decreased in mice with sleep disruption, while the placental weight showed no change between the two groups (Fig. 6k, l, Additional file 9: Fig. S9). Both SR9009, a Rev-erbα agonist and IL-6 neutralized antibody could alleviate the adverse effect of sleep disruption on decidualization and pregnancy outcomes (Fig. 6b–l, Additional file 9: Fig. S9). These results suggested that Rev-erbα—IL-6/IL-6R-PR-C/EBPβ axis affected by sleep played vital roles in decidualization and pregnancy maintenance.

## Discussion

Circadian rhythm makes the body adapt to the environmental changes for survival. The light–dark cycle and the sleep–wake cycle are two main synchronizers of clock, whose disruption can be induced by an irregular light–dark cycle (such as jet-lag, shift working, sleep disorder, and so on) and increase the risk of gastrointestinal disease, cardiovascular disease, diabetes and metabolic disturbances [17, 33, 34]. Recently, numerous studies supplied evidences to support the association between sleep disturbance and adverse reproductive outcomes [3, 4], but the biological mechanisms underlying this connection remain unclear. In this study, 22.37% (17/76 patients in our small clinical surveys) patients with infertility or miscarriage have symptoms of sleep disturbance. Decreased Rev-erbα expression and deficient decidualization of ESCs were observed in early pregnancy loss under sleep disturbance both in human beings and in mice. These results suggested that Rev-erbα might be a link between disordered circadian rhythm induced by sleep disturbance and adverse reproductive outcomes. Moreover, Rev-erbα could regulate decidualization via IL-6/IL-6R-PR-C/EBPβ axis. Mice with sleep disturbance indeed displayed low implantation number and higher abortion rate. This effect of sleep disruption on decidualization and pregnancy outcomes in mice could be alleviated by Rev-erbα agonist and IL-6 neutralized antibody, which might be novel therapeutic targets for infertility and miscarriages induced by sleep disturbance.

It has been reported that SR9009, a Rev-erbα agonist, played roles in inhibiting autophagy and inflammation and were considered to be a potential therapeutic drug for tumor and colitis [17, 35]. We demonstrated that knockdown of *Rev-erbα* promoted the production of proinflammatory factor such as IL-6 in ESCs. Moreover, in vivo experiments exhibited that SR9009 could decrease the production of IL-6 and ameliorate pregnancy outcome of mice with sleep disturbance. IL-6 binds with IL-6R to activate intracellular signaling pathways through both classic and trans-signaling. Blockade of the IL-6/IL-6R signaling pathway has become a promising target for the therapy of cancers and inflammatory autoimmune diseases [36, 37]. In our study, IL-6 neutralized antibody could alleviate adverse pregnancy outcomes of mice with sleep disturbance. Tocilizumab is a recombinant humanized IL-6R neutralizing antibody, which prevents binding of IL-6 to the IL-6R. And it could alleviate defective decidualization in hESCs with *Rev-erbα* knockdown. Therefore, treatments targeting Rev-erbα and IL-6/IL-6R signaling pathway might be effective means to ameliorate pregnancy for human miscarriages with sleep disturbance.

Circadian genes expression could be affected by many factors such as inflammation and hormone [17, 26]. Previous researches suggested that circadian rhythm disruption caused the increased inflammatory cytokines expression [38]. We also reported that *Rev-erbα* knockdown increased the expression of proinflammatory cytokines such as IL-1β, IL-6 and TNF-α in hESCs [27]. In this study, we found that the proinflammatory cytokine IL-6 was the target of Rev-erbα, and interestingly, IL-6 could also repress Rev-erbα expression. It was postulated that there was a feedback loop between Rev-erbα and proinflammatory cytokines as circadian rhythm disruption increased proinflammatory cytokine expression to affect decidualization, while the increased proinflammatory cytokine could further amplify this effect. However, it is still unclear whether the proinflammatory environment or the decreased Rev-erbα expression comes first after sleep disruption.

Pregnancy is a complex physiological process. Sleep disruption affects not only decidualization, but also implantation [7, 8, 39]. In line with the previous study, mice with sleep disturbance displayed the decreased number implantation. The quality of embryo and uterine receptivity are two major determinants for successful implantation. Sleep disruption increases inflammatory level and oxidative stress, which could have negative effects not only on decidualization and subsequent uterine receptivity, but also on oocyte quality and embryo development [38, 40, 41]. To further address the impact of sleep disturbance on fertilized eggs, we will transfer fertilized eggs from parents with sleep disturbance to the oviducts of female mice with normal sleep using in vitro fertilization- embryo transfer methods to detect pregnancy outcomes in the future. In addition, the cross talk between embryo and uterine luminal epithelium is critical for implantation process, and the function of uterine luminal epithelium is regulated by estrogen, progesterone, and factors secreted by ESCs[42–44]. Abnormal hormone secretion and ESCs function induced by sleep disruption may destroy the function of uterine luminal epithelium cells and the stromal—epithelial communication, which might cause adverse pregnancy outcomes. Therefore, adverse pregnancy outcomes induced by sleep disruption might be caused by many factors, not only decidualization.

## Conclusions

**Fig. 7:**
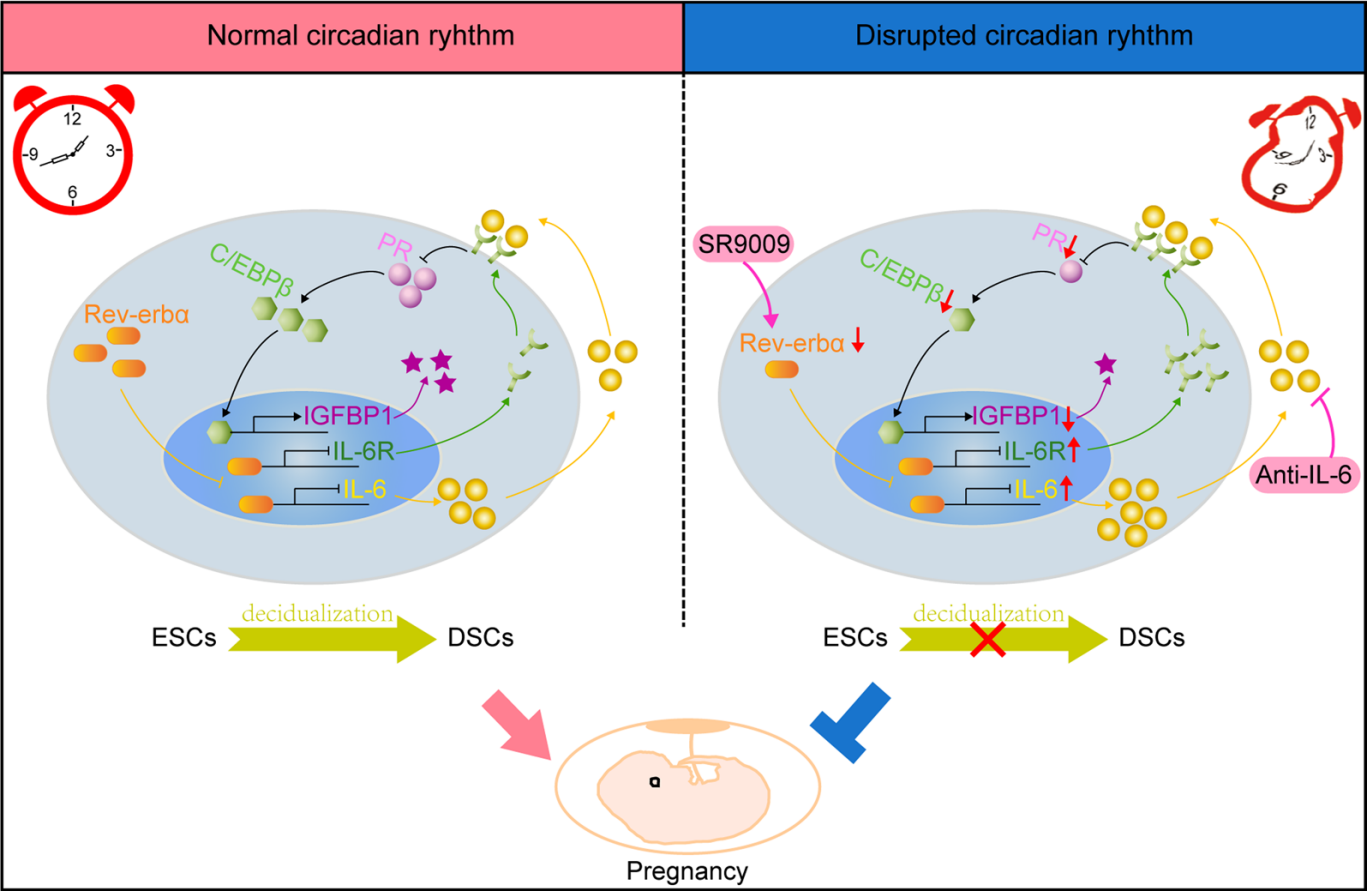
Schematic diagram showing the roles of circadian rhythm in decidualization and pregnancy. The expression of Rev-erbα depends on normal sleep, which is essential for pregnant establishment and maintenance. Rev-erbα, as a transcription factor, directly repressed IL-6/IL-6R expression via binding their promoter region. IL-6/IL-6R axis could suppress the expression of C/EBPβ and its target IGFBP1, the marker of decidualization, by inhibiting PR expression. Sleep disturbance suppressed the expression of Rev-erbα in ESCs, which induced deficient decidualization via the disequilibrated IL-6/IL-6R-PR-C/EBPβ signal axis. Administration with Rev-erbα agonist SR9009 and IL-6 neutralized antibody could both alleviate the defective decidualization and adverse pregnancy outcomes induced by sleep disturbance.

In summary, the expression of Rev-erbα depends on normal sleep, which is essential for pregnant establishment and maintenance. Rev-erbα, as a transcription factor, directly repressed IL-6/IL-6R expression via binding their promoter region. IL-6/IL-6R axis could suppress the expression of C/EBPβ and its target molecules IGFBP1, a marker of decidualization, by inhibiting PR expression. Sleep disturbance suppressed the expression of Rev-erbα in ESCs, which induced deficient decidualization via the disequilibrated IL-6-PR-C/EBPβ signal axis. Administration with Rev-erbα agonist SR9009 and IL-6 neutralized antibody could both alleviate the defective decidualization and adverse pregnancy outcomes induced by sleep disturbance (Fig. 7) These results indicated that Rev-erbα might be a connector between sleep disruption and pregnancy. Our study might provide potential therapeutic targets for adverse pregnancy outcomes induced by circadian rhythm disruption.

## Supplementary Information

**Additional file 1: Fig. S1. **Circadian rhythm of clock genes in liver of mice. **a**–**c** Relative mRNA level of clock genes (*Rev-erbα*, *Rev-erbβ*, *Bmal1*) in liver of mice with normal sleep in 24 h. White box represented light-on time. Black box represented light-off time. The time of light on referred to ZT0. Data represented Mean±SEM.

**Additional file 2: Fig. S2.** Downregulated Rev-erbα expression in ESCs of mice and human with sleep disruption. **a** (left) Immunofluorescence for Rev-erbα and Vimentin in uterus of mice with normal sleep or sleep disturbance at ZT3. (right) The relative mean gray value of Rev-erbα in Vimentin^+^ ESCs from mice with normal sleep or sleep disturbance at ZT3. **b** (left) Immunofluorescence for Rev-erbα and Vimentin in uterus of mice with normal sleep or sleep disturbance at ZT9. (right) The relative mean gray value of Rev-erbα in Vimentin^+^ ESCs from mice with normal sleep or sleep disturbance at ZT9. **c** (left) Immunofluorescence for Rev-erbα and Vimentin in endometrial tissues of human with normal sleep or sleep disturbance. (right) The relative mean gray value of Rev-erbα in Vimentin^+^ ESCs from human with normal sleep or sleep disturbance. mNS represented mouse with normal sleep. mSD represented mouse with sleep disturbance. hNS represented human with normal sleep. hSD represented human with sleep disturbance. The time of light on referred to ZT0. Data represented Mean±SEM. Statistical analysis was performed using Student’s *t*‐test. ***P<0.001, ****P<0.0001.

**Additional file 3: Fig. S3. **Rev-erbα expression in murine uterine tissue at different gestation period. **a** Immunofluorescence for Rev-erbα and Vimentin in murine uterine tissue at different gestation period. **b** The protein level of Rev-erbα in murine uterine tissue at different gestation period. Relative protein levels were normalized to β-Actin. Data represented Mean±SEM. Statistical analysis was performed using Student’s *t*‐test. ***P<0.001, ****P<0.0001.

**Additional file 4: Fig. S4.** Rev-erbα expression in hESCs and hDSCs. **a** The protein level of Rev-erbα in hESCs and hDSCs from human with normal sleep. **b** Relative mRNA level of clock genes (*Rev-erbβ*, *Bmal1*, *Clock*) in hDSCs from normal pregnancies with normal sleep and miscarriages with sleep disturbance. **c** Immunofluorescence for decidual tissues from human normal pregnancies with normal sleep and miscarriages with sleep disturbance. hNP represented human with normal pregnancy and normal sleep. hMis represented human with miscarriage and sleep disturbance. Data represented Mean±SEM. Statistical analysis was performed using Student’s *t*‐test. *P<0.05, **P<0.01, ***P<0.001.

**Additional file 5: Fig. S5.** Downregulated Rev-erbα and Wnt4 expression in DSCs of mice with sleep disturbance after artificial decidualization. **a** (up) Immunofluorescence for Rev-erbα and Vimentin in oil-injected lateral uterus of mice with normal or sleep disturbance. (down) The relative mean gray value of Rev-erbα in Vimentin^+^ DSCs from mice with normal sleep or sleep disturbance. **b** (up) Immunofluorescence for Wnt4 and Vimentin in oil-injected lateral uterus of mice with normal or sleep disturbance. (down) The relative mean gray value of Wnt4 in Vimentin^+^DSCs from mice with normal sleep or sleep disturbance. mNS represented mouse with normal sleep. mSD represented mouse with sleep disturbance. Data represented Mean±SEM. Statistical analysis was performed using Student’s *t*‐test. ****P<0.001.

**Additional file 6: Fig. S6.** *Rev-erbα* knockdown induced defective decidualization in mESCs**. **SR9009 alleviated the defective decidualization induced by *Rev-erbα* knockdown in mESCs. Relative protein levels were normalized to β-Tubulin. Data represented Mean±SEM. Statistical analysis was performed using Student’s *t*‐test. *P<0.05, ***P<0.001.

**Additional file 7: Fig. S7.** *Rev-erbα* knockdown downregulated PR and C/EBPβ expression in mESCs. **a **The protein level of PR and C/EBPβ in mESCs with or without *Rev-erbα* knockdown. Relative protein levels were normalized to β-Tubulin. **b **The protein level of PR and C/EBPβ in mESCs with or without *PGR* knockdown. Relative protein levels were normalized to β-Tubulin. **c** The protein level of C/EBPβ and Wnt4 in mESCs with or without *C/EBPβ* knockdown. Relative protein levels were normalized to β-Tubulin. Data represented Mean±SEM. Statistical analysis was performed using Student’s *t*‐test. *P<0.05, **P<0.01.

**Additional file 8: Fig. S8.** Rev-erbα regulated decidualization via IL-6-PR-C/EBPβ axis in mESCs. **a** The protein level of IL-6 and IL-6R in mESCs with or without *Rev-erbα* knockdown. Relative protein levels were normalized to β-Tubulin. **b** The protein level of PR, C/EBPβ and Wnt4 in mESCs with IL-6 stimulation. Relative protein levels were normalized to β-Tubulin. **c** IL-6 neutralized antibody (anti-IL-6) reversed the decreased PR, C/EBPβ and Wnt4 expression in mESCs with *Rev-erbα* knockdown. Relative protein levels were normalized to β-Tubulin. Data represented Mean±SEM. Statistical analysis was performed using Student’s *t*‐test. *P<0.05, **P<0.01, ***P<0.001, ****P<0.0001.

**Additional file 9: Fig. S9. **Representative pictures of embryos and placentas of mice with normal sleep and those of mice with sleep disturbance under SR9009 or IL-6 neutralized antibody (anti-IL-6) treatment.

## Data Availability

All data presented in this study are included in this published article and its supplementary information files.

## Abbreviations

SCN: Suprachiasmatic nucleus
Bmal1: Brain and muscle ARNT-like protein
Clock: Circadian locomotor output cycles kaput
hESCs: Human endometrial stromal cells
hDSCs: Human decidual stromal cells
IL-6: Interlukin 6
PR: Progesterone receptor
IGFBP1: Insulin-like growth factor binding protein 1
C/EBPβ: CCAAT/enhancer-binding protein β
QPCR: Quantitative Real-time PCR
PSQI: Pittsburgh sleep quality index
ChIP-PCR: Chromatin immunoprecipitation-polymerase chain reaction
HE: Immunofluorescence and hematoxylin–eosin
