# Supplementary material for: Circadian gene Rev-erbα influenced by sleep conduces to pregnancy by promoting endometrial decidualization via IL-6-PR-C/EBPβ axis
Source: J Biomed Sci. 2022 Nov 24;29:101. doi: 10.1186/s12929-022-00884-1 (PMC9685872; doi:10.1186/s12929-022-00884-1)
Supplement: Supplementary file 1 — Additional file 1: Fig. S1. Circadian rhythm of clock genes in liver of mice. a–c Relative mRNA level of clock genes (Rev-erbα, Rev-erbβ, Bmal1) in liver of mice with normal sleep in 24 h. White box represented light-on time. Black box represented light-off time. The time of light on referred to ZT0. Data represented Mean±SEM. [file 12929_2022_884_MOESM1_ESM.docx]

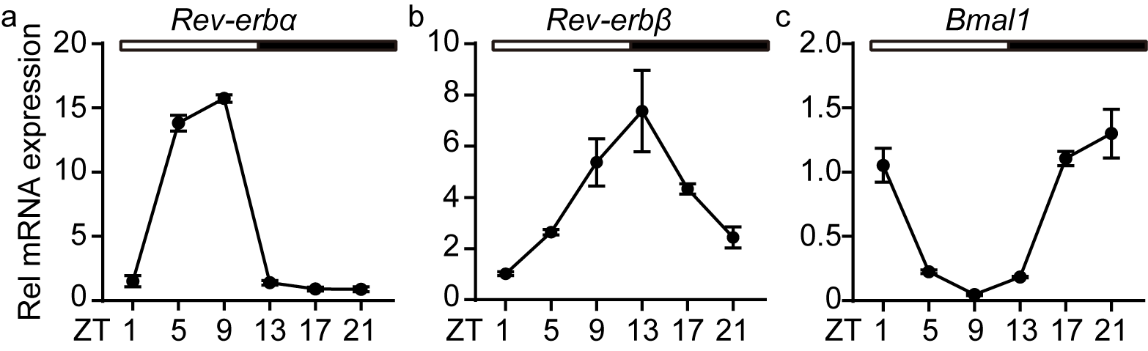


**Fig. S1 Circadian rhythm of clock genes in liver of mice.** **a-c** Relative mRNA level of clock genes (*Rev-erbα*, *Rev-erbβ*, *Bmal1*) in liver of mice with normal sleep in 24 hours. White box represented light-on time. Black box represented light-off time. The time of light on referred to ZT0. Data represented Mean±SEM.
