# Supplementary material for: Circadian gene Rev-erbα influenced by sleep conduces to pregnancy by promoting endometrial decidualization via IL-6-PR-C/EBPβ axis
Source: J Biomed Sci. 2022 Nov 24;29:101. doi: 10.1186/s12929-022-00884-1 (PMC9685872; doi:10.1186/s12929-022-00884-1)
Supplement: Supplementary file 2 — Additional file 2: Fig. S2. Downregulated Rev-erbα expression in ESCs of mice and human with sleep disruption. a (left) Immunofluorescence for Rev-erbα and Vimentin in uterus of mice with normal sleep or sleep disturbance at ZT3. (right) The relative mean gray value of Rev-erbα in Vimentin+ ESCs from mice with normal sleep or sleep disturbance at ZT3. b (left) Immunofluorescence for Rev-erbα and Vimentin in uterus of mice with normal sleep or sleep disturbance at ZT9. (right) The relative mean gray value of Rev-erbα in Vimentin+ ESCs from mice with normal sleep or sleep disturbance at ZT9. c (left) Immunofluorescence for Rev-erbα and Vimentin in endometrial tissues of human with normal sleep or sleep disturbance. (right) The relative mean gray value of Rev-erbα in Vimentin+ ESCs from human with normal sleep or sleep disturbance. mNS represented mouse with normal sleep. mSD represented mouse with sleep disturbance. hNS represented human with normal sleep. hSD represented human with sleep disturbance. The time of light on referred to ZT0. Data represented Mean±SEM. Statistical analysis was performed using Student’s t‐test. ***P<0.001, ****P<0.0001. [file 12929_2022_884_MOESM2_ESM.docx]

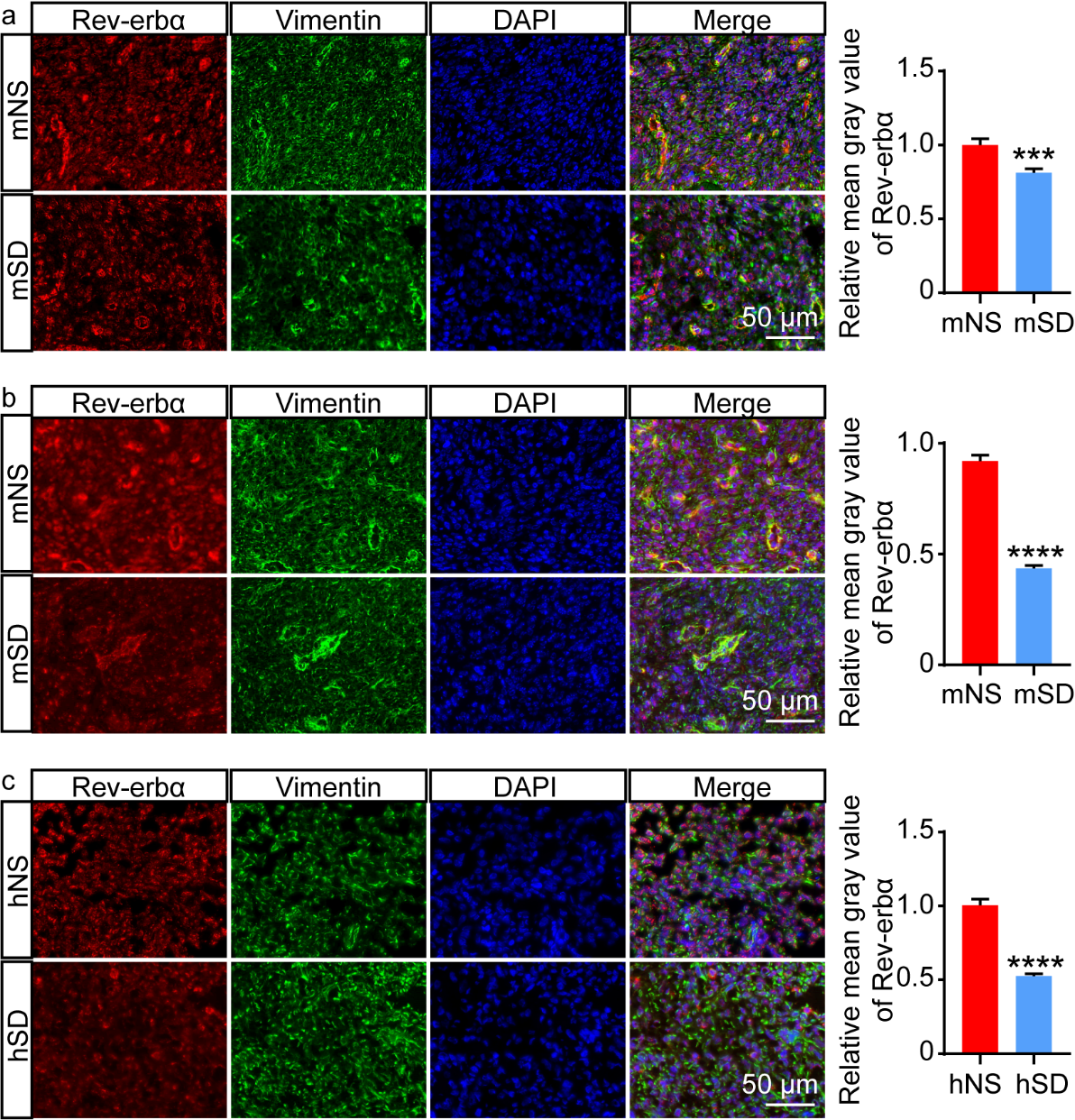


**Fig.S2 Downregulated Rev-erbα expression** **in ESCs of mice and human with sleep disruption. a** (left) Immunofluorescence for Rev-erbα and Vimentin in uterus of mice with normal sleep or sleep disturbance at ZT3. (right) The relative mean gray value of Rev-erbα in Vimentin^+^ ESCs from mice with normal sleep or sleep disturbance at ZT3. **b** (left) Immunofluorescence for Rev-erbα and Vimentin in uterus of mice with normal sleep or sleep disturbance at ZT9. (right) The relative mean gray value of Rev-erbα in Vimentin^+^ ESCs from mice with normal sleep or sleep disturbance at ZT9. **c** (left) Immunofluorescence for Rev-erbα and Vimentin in endometrial tissues of human with normal sleep or sleep disturbance. (right) The relative mean gray value of Rev-erbα in Vimentin^+^ ESCs from human with normal sleep or sleep disturbance. mNS represented mouse with normal sleep. mSD represented mouse with sleep disturbance. hNS represented human with normal sleep. hSD represented human with sleep disturbance. The time of light on referred to ZT0. Data represented Mean±SEM. Statistical analysis was performed using Student’s *t*‐test. ***P<0.001, ****P<0.0001.
