# Supplementary material for: Circadian gene Rev-erbα influenced by sleep conduces to pregnancy by promoting endometrial decidualization via IL-6-PR-C/EBPβ axis
Source: J Biomed Sci. 2022 Nov 24;29:101. doi: 10.1186/s12929-022-00884-1 (PMC9685872; doi:10.1186/s12929-022-00884-1)
Supplement: Supplementary file 3 — Additional file 3: Fig. S3. Rev-erbα expression in murine uterine tissue at different gestation period. a Immunofluorescence for Rev-erbα and Vimentin in murine uterine tissue at different gestation period. b The protein level of Rev-erbα in murine uterine tissue at different gestation period. Relative protein levels were normalized to β-Actin. Data represented Mean±SEM. Statistical analysis was performed using Student’s t‐test. ***P<0.001, ****P<0.0001. [file 12929_2022_884_MOESM3_ESM.docx]

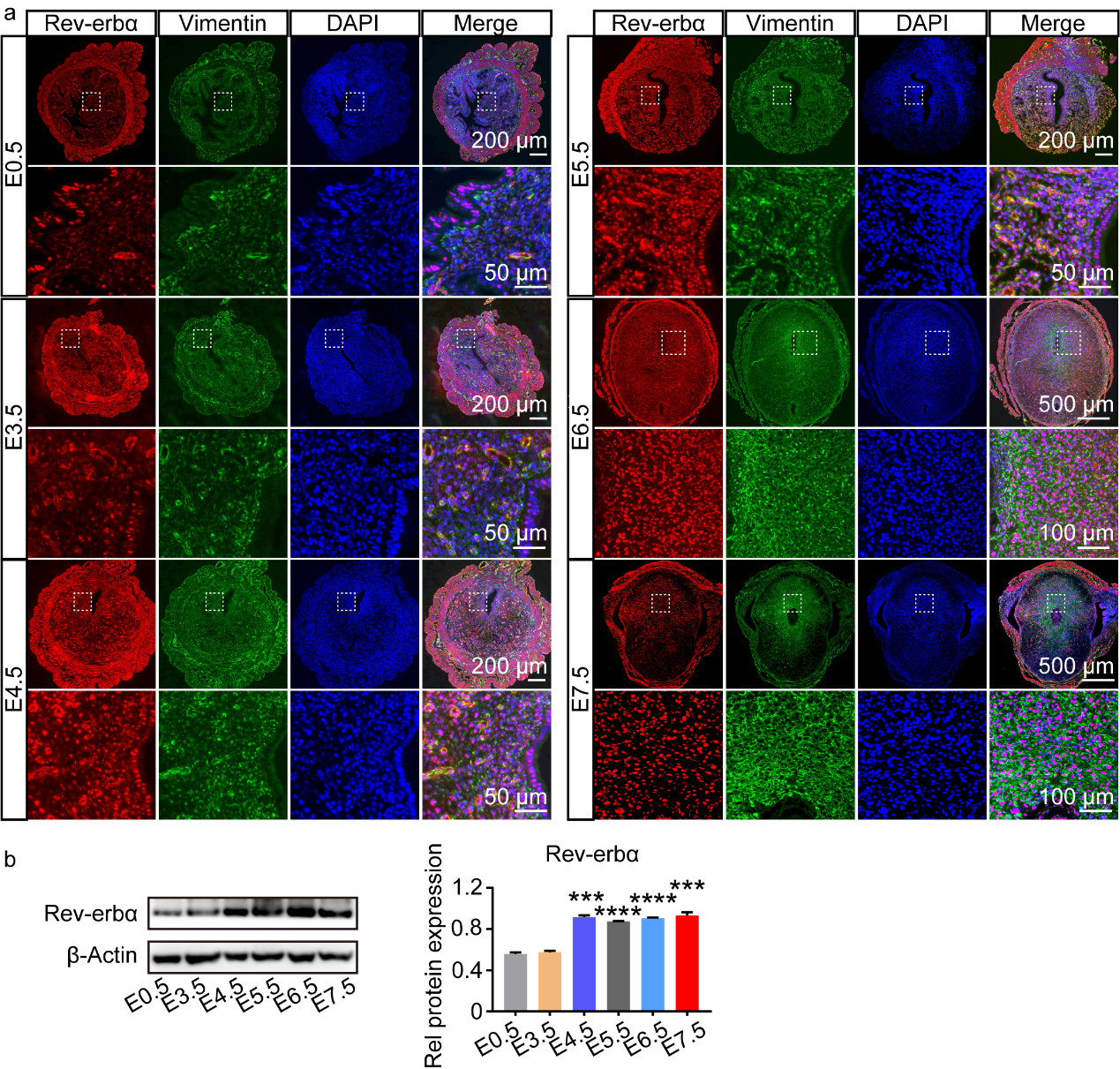


**Fig.S3 Rev-erbα expression in murine uterine tissue at different gestation period. a** Immunofluorescence for Rev-erbα and Vimentin in murine uterine tissue at different gestation period. **b** The protein level of Rev-erbα in murine uterine tissue at different gestation period. Relative protein levels were normalized to β-Actin. Data represented Mean±SEM. Statistical analysis was performed using Student’s *t*‐test. ***P<0.001, ****P<0.0001.
