# Supplementary material for: Circadian gene Rev-erbα influenced by sleep conduces to pregnancy by promoting endometrial decidualization via IL-6-PR-C/EBPβ axis
Source: J Biomed Sci. 2022 Nov 24;29:101. doi: 10.1186/s12929-022-00884-1 (PMC9685872; doi:10.1186/s12929-022-00884-1)
Supplement: Supplementary file 4 — Additional file 4: Fig. S4. Rev-erbα expression in hESCs and hDSCs. a The protein level of Rev-erbα in hESCs and hDSCs from human with normal sleep. b Relative mRNA level of clock genes (Rev-erbβ, Bmal1, Clock) in hDSCs from normal pregnancies with normal sleep and miscarriages with sleep disturbance. c Immunofluorescence for decidual tissues from human normal pregnancies with normal sleep and miscarriages with sleep disturbance. hNP represented human with normal pregnancy and normal sleep. hMis represented human with miscarriage and sleep disturbance. Data represented Mean±SEM. Statistical analysis was performed using Student’s t‐test. *P<0.05, **P<0.01, ***P<0.001. [file 12929_2022_884_MOESM4_ESM.docx]

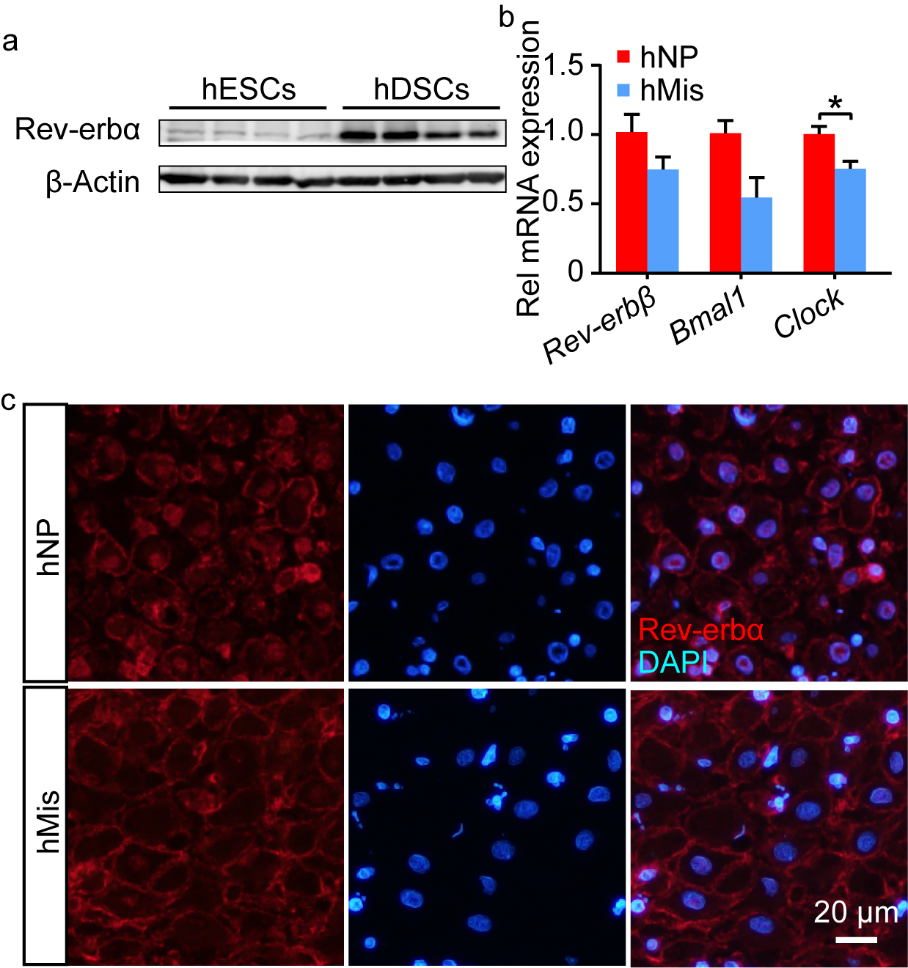


**Fig. S4 Rev-erbα expression in hESCs and hDSCs. a** The protein level of Rev-erbα in hESCs and hDSCs from human with normal sleep. **b** Relative mRNA level of clock genes (*Rev-erbβ*, *Bmal1*, *Clock*) in hDSCs from normal pregnancies with normal sleep and miscarriages with sleep disturbance. **c** Immunofluorescence for decidual tissues from human normal pregnancies with normal sleep and miscarriages with sleep disturbance. hNP represented human with normal pregnancy and normal sleep. hMis represented human with miscarriage and sleep disturbance. Data represented Mean±SEM. Statistical analysis was performed using Student’s *t*‐test. *P<0.05, **P<0.01, ***P<0.001.
