# Supplementary material for: Circadian gene Rev-erbα influenced by sleep conduces to pregnancy by promoting endometrial decidualization via IL-6-PR-C/EBPβ axis
Source: J Biomed Sci. 2022 Nov 24;29:101. doi: 10.1186/s12929-022-00884-1 (PMC9685872; doi:10.1186/s12929-022-00884-1)
Supplement: Supplementary file 5 — Additional file 5: Fig. S5. Downregulated Rev-erbα and Wnt4 expression in DSCs of mice with sleep disturbance after artificial decidualization. a (up) Immunofluorescence for Rev-erbα and Vimentin in oil-injected lateral uterus of mice with normal or sleep disturbance. (down) The relative mean gray value of Rev-erbα in Vimentin+ DSCs from mice with normal sleep or sleep disturbance. b (up) Immunofluorescence for Wnt4 and Vimentin in oil-injected lateral uterus of mice with normal or sleep disturbance. (down) The relative mean gray value of Wnt4 in Vimentin+DSCs from mice with normal sleep or sleep disturbance. mNS represented mouse with normal sleep. mSD represented mouse with sleep disturbance. Data represented Mean±SEM. Statistical analysis was performed using Student’s t‐test. ****P<0.001. [file 12929_2022_884_MOESM5_ESM.docx]

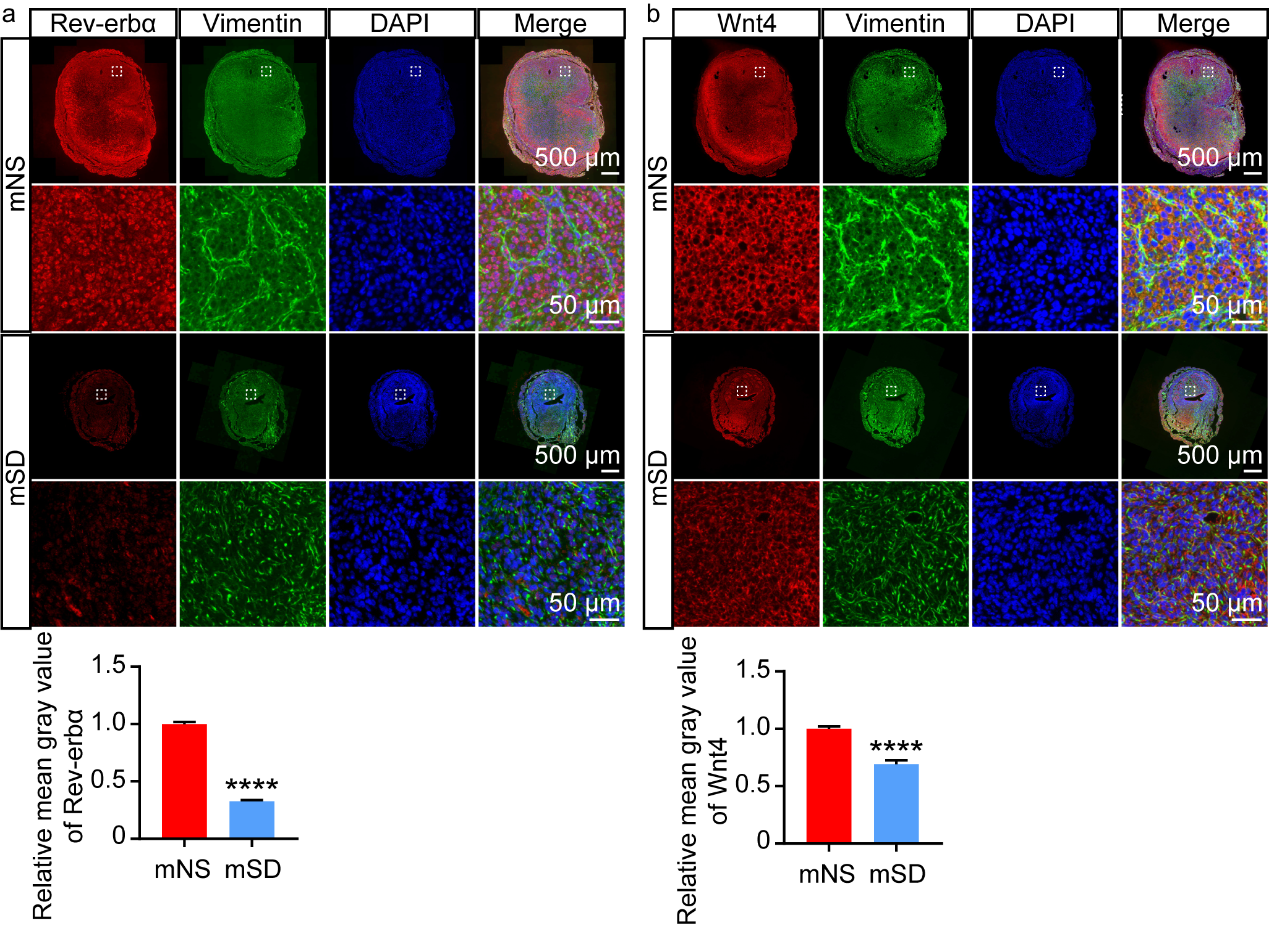


**Fig. S5 Downregulated Rev-erbα and Wnt4 expression in DSCs of mice with sleep disturbance** **after artificial decidualization. a** (up) Immunofluorescence for Rev-erbα and Vimentin in oil-injected lateral uterus of mice with normal or sleep disturbance. (down) The relative mean gray value of Rev-erbα in Vimentin^+^ DSCs from mice with normal sleep or sleep disturbance. **b** (up) Immunofluorescence for Wnt4 and Vimentin in oil-injected lateral uterus of mice with normal or sleep disturbance. (down) The relative mean gray value of Wnt4 in Vimentin^+^DSCs from mice with normal sleep or sleep disturbance. mNS represented mouse with normal sleep. mSD represented mouse with sleep disturbance. Data represented Mean±SEM. Statistical analysis was performed using Student’s t‐test. ****P<0.001.
