# Supplementary material for: Circadian gene Rev-erbα influenced by sleep conduces to pregnancy by promoting endometrial decidualization via IL-6-PR-C/EBPβ axis
Source: J Biomed Sci. 2022 Nov 24;29:101. doi: 10.1186/s12929-022-00884-1 (PMC9685872; doi:10.1186/s12929-022-00884-1)
Supplement: Supplementary file 6 — Additional file 6: Fig. S6. Rev-erbα knockdown induced defective decidualization in mESCs. SR9009 alleviated the defective decidualization induced by Rev-erbα knockdown in mESCs. Relative protein levels were normalized to β-Tubulin. Data represented Mean±SEM. Statistical analysis was performed using Student’s t‐test. *P<0.05, ***P<0.001. [file 12929_2022_884_MOESM6_ESM.docx]

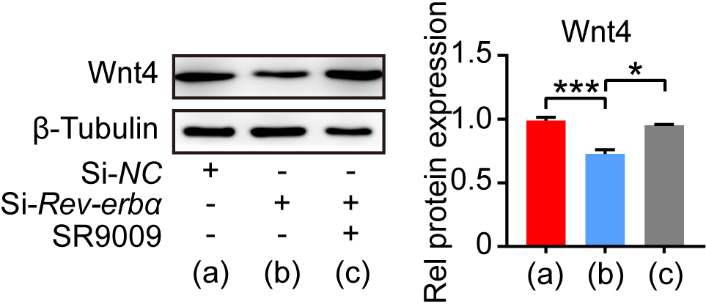


**Fig. S6 *Rev-erbα* knockdown induced defective decidualization in mESCs.** SR9009 alleviated the defective decidualization induced by *Rev-erbα* knockdown in mESCs. Relative protein levels were normalized to β-Tubulin. Data represented Mean±SEM. Statistical analysis was performed using Student’s t‐test. *P<0.05, ***P<0.001.
