# Supplementary material for: Circadian gene Rev-erbα influenced by sleep conduces to pregnancy by promoting endometrial decidualization via IL-6-PR-C/EBPβ axis
Source: J Biomed Sci. 2022 Nov 24;29:101. doi: 10.1186/s12929-022-00884-1 (PMC9685872; doi:10.1186/s12929-022-00884-1)
Supplement: Supplementary file 7 — Additional file 7: Fig. S7. Rev-erbα knockdown downregulated PR and C/EBPβ expression in mESCs. a The protein level of PR and C/EBPβ in mESCs with or without Rev-erbα knockdown. Relative protein levels were normalized to β-Tubulin. b The protein level of PR and C/EBPβ in mESCs with or without PGR knockdown. Relative protein levels were normalized to β-Tubulin. c The protein level of C/EBPβ and Wnt4 in mESCs with or without C/EBPβ knockdown. Relative protein levels were normalized to β-Tubulin. Data represented Mean±SEM. Statistical analysis was performed using Student’s t‐test. *P<0.05, **P<0.01. [file 12929_2022_884_MOESM7_ESM.docx]

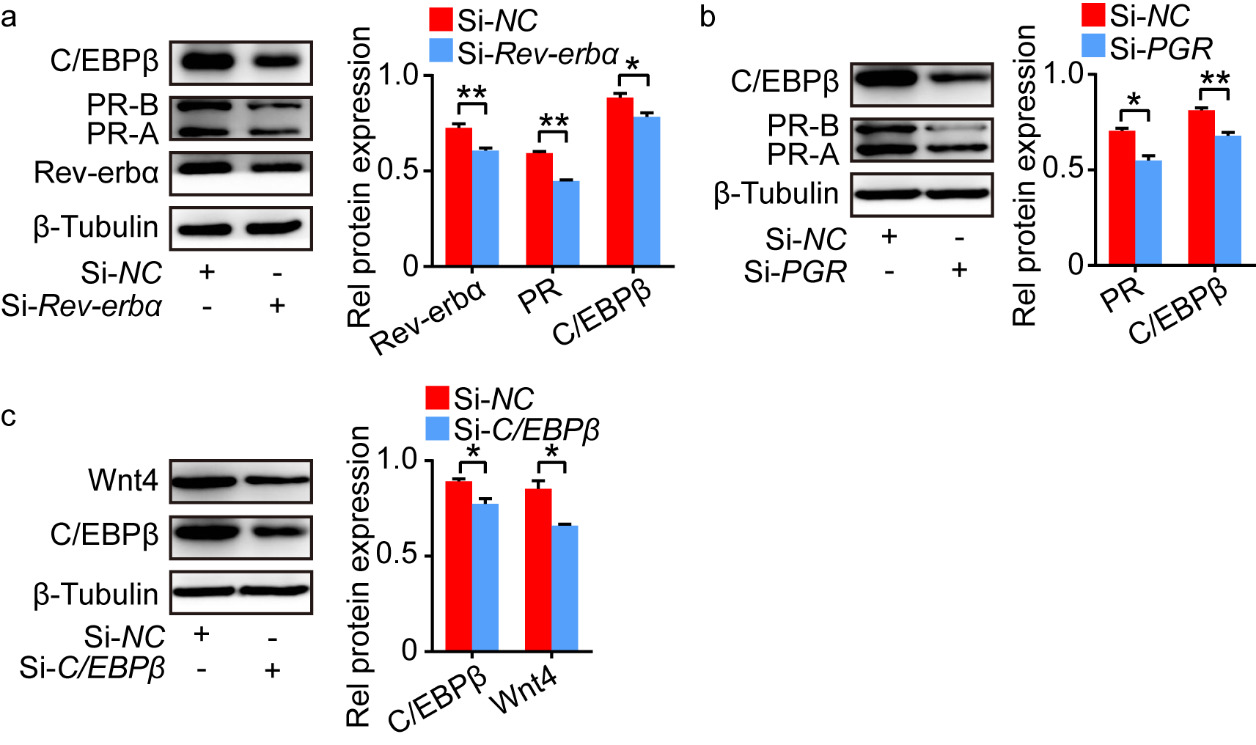


**Fig. S7 *Rev-erbα* knockdown downregulated PR and C/EBPβ expression in mESCs. a** The protein level of PR and C/EBPβ in mESCs with or without *Rev-erbα* knockdown. Relative protein levels were normalized to β-Tubulin. **b** The protein level of PR and C/EBPβ in mESCs with or without *PGR* knockdown. Relative protein levels were normalized to β-Tubulin. **c** The protein level of C/EBPβ and Wnt4 in mESCs with or without *C/EBPβ* knockdown. Relative protein levels were normalized to β-Tubulin. Data represented Mean±SEM. Statistical analysis was performed using Student’s *t*‐test. *P<0.05, **P<0.01.
