# Supplementary material for: Circadian gene Rev-erbα influenced by sleep conduces to pregnancy by promoting endometrial decidualization via IL-6-PR-C/EBPβ axis
Source: J Biomed Sci. 2022 Nov 24;29:101. doi: 10.1186/s12929-022-00884-1 (PMC9685872; doi:10.1186/s12929-022-00884-1)
Supplement: Supplementary file 8 — Additional file 8: Fig. S8. Rev-erbα regulated decidualization via IL-6-PR-C/EBPβ axis in mESCs. a The protein level of IL-6 and IL-6R in mESCs with or without Rev-erbα knockdown. Relative protein levels were normalized to β-Tubulin. b The protein level of PR, C/EBPβ and Wnt4 in mESCs with IL-6 stimulation. Relative protein levels were normalized to β-Tubulin. c IL-6 neutralized antibody (anti-IL-6) reversed the decreased PR, C/EBPβ and Wnt4 expression in mESCs with Rev-erbα knockdown. Relative protein levels were normalized to β-Tubulin. Data represented Mean±SEM. Statistical analysis was performed using Student’s t‐test. *P<0.05, **P<0.01, ***P<0.001, ****P<0.0001. [file 12929_2022_884_MOESM8_ESM.docx]

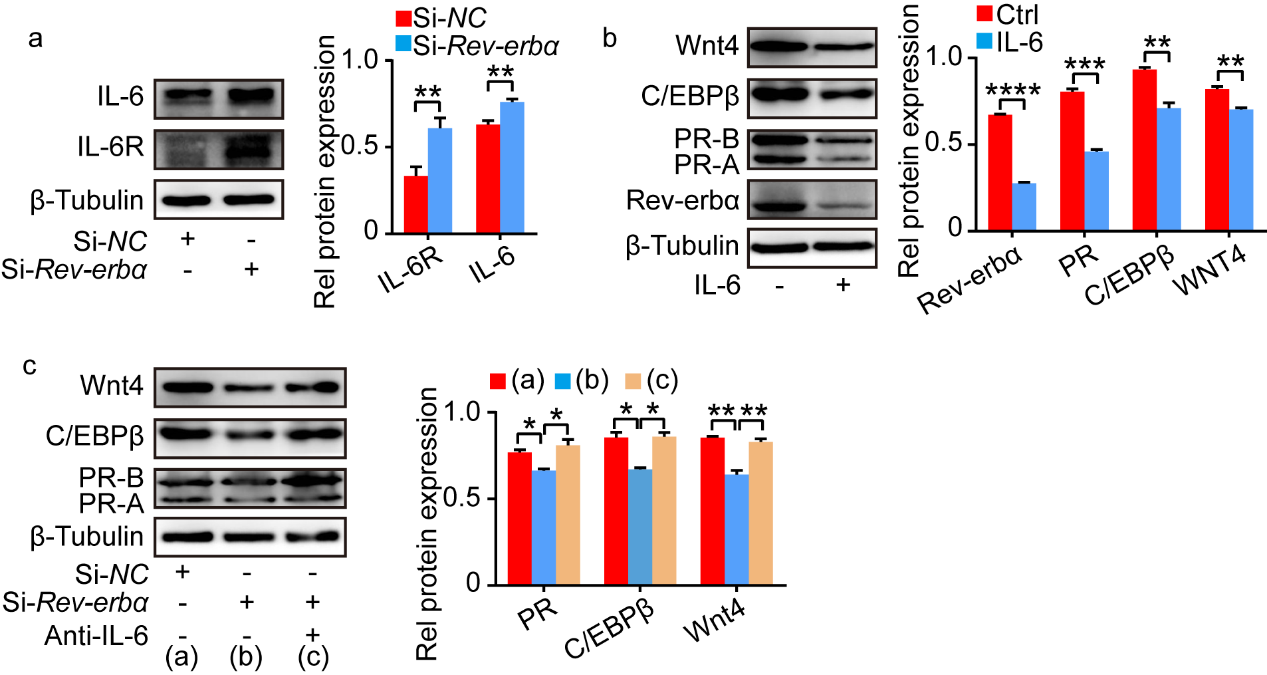


**Fig. S8 Rev-erbα regulated decidualization via IL-6-PR-C/EBPβ axis in mESCs. a** The protein level of IL-6 and IL-6R in mESCs with or without *Rev-erbα* knockdown. Relative protein levels were normalized to β-Tubulin. **b** The protein level of PR, C/EBPβ and Wnt4 in mESCs with IL-6 stimulation. Relative protein levels were normalized to β-Tubulin. **c** IL-6 neutralized antibody (anti-IL-6) reversed the decreased PR, C/EBPβ and Wnt4 expression in mESCs with *Rev-erbα* knockdown. Relative protein levels were normalized to β-Tubulin. Data represented Mean±SEM. Statistical analysis was performed using Student’s *t*‐test. *P<0.05, **P<0.01, ***P<0.001, ****P<0.0001.
