# Supplementary material for: Circadian gene Rev-erbα influenced by sleep conduces to pregnancy by promoting endometrial decidualization via IL-6-PR-C/EBPβ axis
Source: J Biomed Sci. 2022 Nov 24;29:101. doi: 10.1186/s12929-022-00884-1 (PMC9685872; doi:10.1186/s12929-022-00884-1)
Supplement: Supplementary file 9 — Additional file 9: Fig. S9. Representative pictures of embryos and placentas of mice with normal sleep and those of mice with sleep disturbance under SR9009 or IL-6 neutralized antibody (anti-IL-6) treatment. [file 12929_2022_884_MOESM9_ESM.docx]

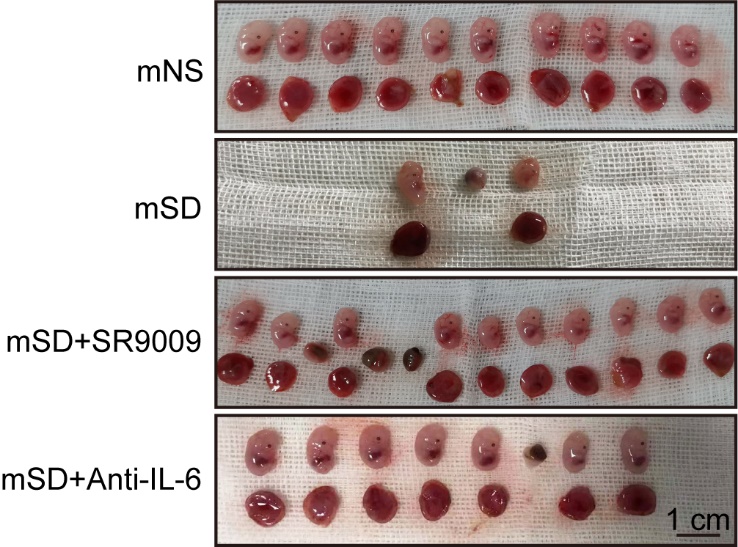


**Fig. S9 Representative pictures of embryos and placentas of mice with normal sleep and those of mice with sleep disturbance under SR9009 or IL-6 neutralized antibody (anti-IL-6) treatment.**
